# Degradation and Defluorination of Per- and Polyfluoroalkyl Substances by Direct Photolysis at 222 nm

**DOI:** 10.1021/acsestwater.3c00274

**Published:** 2023-07-06

**Authors:** Xiaoyue Xin, Juhee Kim, Daniel C. Ashley, Ching-Hua Huang

**Affiliations:** † School of Civil and Environmental Engineering, Georgia Institute of Technology, Atlanta, Georgia 30332, United States; ‡ Department of Chemistry and Biochemistry, Spelman College, Atlanta, Georgia 30314, United States

**Keywords:** PFAS, far-UVC, UV photolysis, defluorination, KrCl* excimer lamp, water treatment

## Abstract

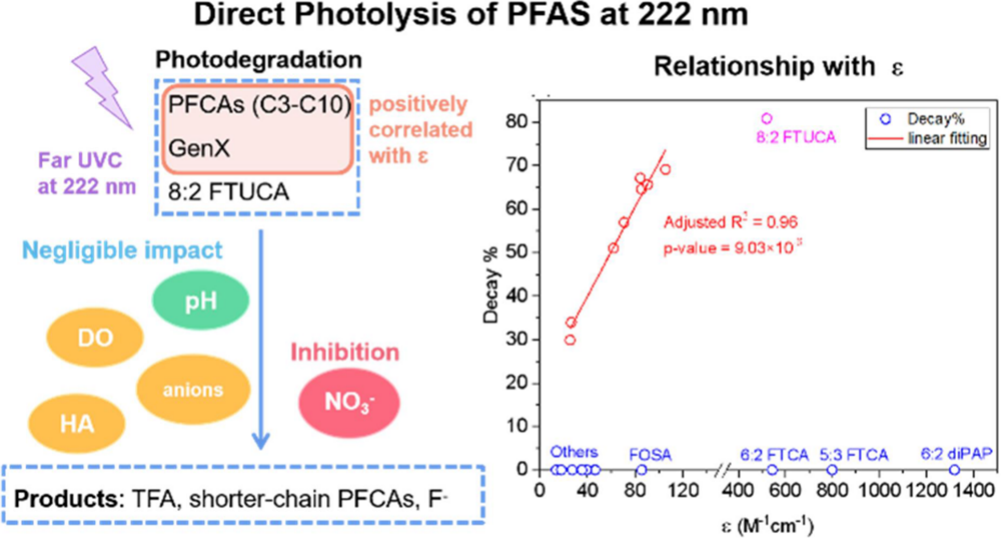

The susceptibility of 19 representative per- and polyfluoroalkyl substances (PFAS) to direct photolysis and defluorination under far-UVC 222 nm irradiation was investigated. Enhanced photolysis occurred for perfluorocarboxylic acids (PFCAs), fluorotelomer unsaturated carboxylic acids (FTUCAs), and GenX, compared to that at conventional 254 nm irradiation on a similar fluence basis, while other PFAS showed minimal decay. For degradable PFAS, up to 81% of parent compound decay (photolysis rate constant (*k*_222 nm_) = 8.19–34.76 L·Einstein^–1^; quantum yield (Φ_222 nm_) = 0.031–0.158) and up to 31% of defluorination were achieved within 4 h, and the major transformation products were shorter-chain PFCAs. Solution pH, dissolved oxygen, carbonate, phosphate, chloride, and humic acids had mild impacts, while nitrate significantly affected PFAS photolysis/defluorination at 222 nm. Decarboxylation is a crucial step of photolytic decay. The slower degradation of short-chain PFCAs than long-chain ones is related to molar absorptivity and may also be influenced by chain-length dependent structural factors, such as differences in p*K*_a_, conformation, and perfluoroalkyl radical stability. Meanwhile, theoretical calculations indicated that the widely proposed HF elimination from the alcohol intermediate (C_*n*_F_2*n*+1_OH) of PFCA is an unlikely degradation pathway due to high activation barriers. These new findings are useful for further development of far-UVC technology for PFAS in water treatment.

## Abstract 2

Far-UVC 222 nm irradiation is capable of degrading groups of PFAS including PFCAs, FTUCAs, and GenX, and the photolysis rate and extent are influenced by molar absorptivity and possibly several chain-length-dependent structural factors.

## 1 Introduction

Per- and polyfluoroalkyl substances (PFAS) are a class of anthropogenic compounds that pose significant health and ecological concerns because of their environmental persistence and toxicity.^1−3^ PFAS have been widely used in many industrial and commercial applications such as water-repellent fabrics, alkaline cleaners, paints, packaged food, carpets, upholstery, shampoos, cookware, firefighting foams, and nonstick products.^4,5^ The disposal of PFAS-containing products and discharge of industrial and municipal wastewaters result in the omnipresence of these compounds in various environmental compartments,^3,6^ tissues of wildlife,^7,8^ and human blood and breast milks.^3,9−11^ In 2016, the U.S. Environmental Protection Agency (USEPA) released a nonenforceable lifetime health advisory level at 70 ppt for perfluorooctanoic acid (PFOA) and perfluorooctane sulfonate (PFOS), individually or combined.^12^ In 2023, the USEPA proposed the enforceable levels (or maximum contamination levels, MCLs) for PFOA and PFOS at 4 ppt, respectively, and four additional PFAS as a mixture: perfluorobutane sulfonic acid (PFBS), perfluorohexane sulfonic acid (PFHxS), perfluorononanoic acid (PFNA), and hexafluoropropylene oxide dimer acid (GenX) using a hazard index approach (HI = 1.0).^13^

Ultraviolet (UV)-based technology is commonly used in water treatment. Common UV setups in water treatment employ low-pressure UV (LPUV) lamps emitting primarily at 254 nm or medium-pressure UV lamps (MPUV) emitting in the range of 200–300 nm.^14−17^ Research has also investigated the application of vacuum UV (VUV), emitting at 185 nm, for the abatement of water contaminants in laboratory settings.^18−20^ Recently, excimer lamps have emerged as a novel alternative UV source that includes a noble gas-halogen dimer generating UV emission when its excited state returns to the ground state.^21^ The krypton chloride (KrCl*) excimer lamps emit narrowly at 222 nm which falls in the so-called far-UVC range of 200–230 nm.^22^ Compared to conventional LPUV lamps, the KrCl* lamps have several advantages, including higher photon energy due to the shorter wavelength, the absence of mercury, minimal harm to exposed human tissues and eyes, and output stability at cold temperatures.^22−24^ The 222 nm irradiation has been demonstrated to be highly effective in inactivating pathogens^25−27^ and degrading some organic pollutants.^28−30^ While the VUV also has the advantage of higher photon energy, the strong light absorption of water at 185 nm (ε = 1.61–1.62 cm^–1^)^31^ substantially limits light penetration. In contrast, the light absorption of water at 222 nm (ε ∼ 0.001 cm^–1^) is only slightly higher than that at 254 nm.^31^ Previous research has shown that most PFAS exhibit negligible to very slow photolysis under LPUV, and hence, photolysis of PFAS has not received much attention.^32−34^ To date, however, research on the potential photolysis of PFAS under 222 nm irradiation is scarce. Moreover, studies on the removal of other PFAS beyond PFOA and PFOS (such as shorter-chains or different structural properties) have been very limited.

The objective of this study was to evaluate the potential photolysis and defluorination of PFAS under far-UVC 222 nm irradiation and assess the involved reaction mechanism. A total of 19 PFAS were chosen for investigation to cover a wide range of chain lengths, functional groups, and structural properties, including perfluorocarboxylic acids (PFCAs, C3-C10), perfluorosulfonic acids (PFSAs, C5-C8), fluorotelomer phosphate diesters (6:2 diPAP), fluorotelomer (unsaturated) carboxylic acids (5:3 FTCA, 6:2 FTCA and 8:2 FTUCA), fluorotelomer sulfonic acids (6:2 FTS), perfluorosulfonamides (FOSA and FHxSA), and per- and polyfluoroethers (HFPO-DA or GenX) (structures shown in Table 1 and Supporting Information (SI) Table S1). First, the suite of PFAS were individually screened for photolysis under 222 nm irradiation, and for those degradable PFAS, transformation products were characterized as well. Comparison with LPUV was also conducted for selected PFAS. Then, PFOA was selected for an in-depth investigation of the effects of reaction conditions on the photolysis, including solution pH, common anions, dissolved oxygen, and real water matrices. Finally, the impacts of PFAS structures on the susceptibility to photolysis at 222 nm irradiation were assessed from compound light absorptivity, individual bond dissociation energy, and activation energy for selected pathways to derive mechanistic insight.

**Table 1:** Chemical Names and Structures of Representative PFAS

Abbreviation	Class	Structure
PFOA	perfluorocarboxylic acid	C_7_F_15_ – COOH
PFOS	perfluorosulfonic acid	C_8_F_17_ – SO_3_H
6:2 FTS	fluorotelomer sulfonic acid	C_5_F_11_ – C_2_F_4_ – SO_3_H
8:2 FTUCA	fluorotelomer unsaturated carboxylic acid	C_7_F_15_ – CF = CH – COOH
6:2 FTCA	fluorotelomer carboxylic acid	C_6_F_13_ – CH_2_ – COOH
FHxSA	perfluorosulfonamide	C_6_F_13_ – SO_2_NH_2_
GenX	per- and polyfluoroether	C_3_F_7_ – O – CF(CF_3_)COOH
6:2 diPAP	fluorotelomer phosphate diester	(C_6_F_13_ – C_2_F_4_ – O – )_2_ – PO_2_H

## 2 Materials and Methods

#### 2.1.1 Chemicals

Information on chemicals and the preparation of PFAS stock solutions is provided in SI Text S1.

#### 2.1.2 Experimental Setup

Reactions were carried out in a sealed quartz reactor with 20 mL solution containing 11.0–35.0 μM PFAS and 5.0 mM NaHCO_3_ (pH 8.5), and the solutions were initially purged by nitrogen gas for 0.5 h to remove dissolved oxygen. The removal of oxygen was conducted in order to compare with another study for advanced reduction processes; however, it was found that the presence or absence of O_2_ had no significant influence on the photolysis rate (see more discussion later). Photolysis experiments employed a collimated beam setup with a KrCl* excimer lamp (Ushio) emitting mainly at 222 nm and a quartz reactor placed underneath (illustrated in SI Figure S1). The lamp was equipped with a filter that removed emissions outside of 222 nm. The reaction solution was continuously stirred magnetically at room temperature. Previous research has applied iodide–iodate actinometry to measure the UV fluence at 222 and 254 nm irradiation, respectively.^35,36^ The UV fluence rate from the excimer lamp setup in this study was determined to be 3.14 × 10^–6^ Einstein·L^–1^·s^–1^ using iodide–iodate actinometry. Most photolysis experiments lasted for 4.0 h. Sample aliquots (10 μL) were drawn from the reactor valve using a syringe at time intervals of 0.5 h into a 2 mL polypropylene (PP) vial, and 1.0 mL methanol was added immediately to quench the reaction and dilute the sample. Similar experiments were also conducted in a real wastewater sample that was the tertiary effluent from a municipal wastewater treatment plant. All sample aliquots were stored at 5 °C until analysis by liquid chromatography time-of-flight mass spectrometry (LC-TOFMS).

For comparison, selected experiments were conducted at 254 nm irradiation in a cylindrical quartz reactor with a quartz plate cover, which is placed in a chamber equipped with an LPUV lamp (G4T5 Hg lamp, Philips TUV4W) (Figure S1). The UV fluence rate was determined to be 2.23 × 10^–6^ Einstein·L^–1^·s^–1^ using iodide–iodate actinometry.^35^ The photolysis experiments lasted for 5.6 h (the same total fluence as that with 222 nm irradiation) and the reaction solution was continuously stirred magnetically at room temperature. The preparation of reaction solutions and monitoring of the reactions followed similar procedures as described above.

All experiments were conducted in duplicates or more. Detailed experimental procedures are provided in SI Text S2.

#### 2.1.3 Sample Analysis

Concentrations of PFAS were determined with an Agilent 1260 Infinity HPLC with a 6230 TOFMS system. Details of the analytical method are provided in SI Text S3. The concentration of fluoride ion (F^–^) released from PFAS was determined by an ion-selective electrode (ISE) (9609BNWP Fluoride Electrode, Thermo Scientific) with limit of detection (LOD) around 0.02 ppm. The accuracy of F^–^ measurement by the ISE was validated by ion chromatography. The potential adsorption of F^–^ to the quartz reactor used in this study was confirmed to be low (<5%, SI Figure S3). The molar absorption coefficient (ε) of individual PFAS was determined by measuring the UV absorbance of PFAS stock solution from 190 to 300 nm using a spectrophotometer (Beckman DU 520).

#### 2.1.4 Theoretical Calculations

Structural effects on PFAS degradation were examined by conducting density functional theory (DFT) calculations. Details of the calculation are provided in SI Text S4. Briefly, all structures were optimized at the B3LYP + D3^37−41^/6-31G**^42,43^ level of theory in the gas phase (the effects of solvation were assessed and found to be minimal on geometry optimization). The electronic energies were then refined with single-point energy calculations employing the larger 6-311++G** basis set.^44,45^ The effect of solvation was included by calculating the free energy of solvation for each compound from single-point energy calculations (at the geometry optimization level of theory) using the SMD implicit solvation model.^46^ Frequency calculations were performed for all optimized structures to both determine the thermochemical corrections necessary to calculate Gibbs free energy and to verify the nature of the stationary points. All transition state structures were confirmed to have one imaginary mode, and all other structures had none. All calculations were performed with the Orca electronic structure package (Version 4.2.1).^47^

#### 2.1.5 Statistical Analysis

Correlation analysis of PFAS decay with several chemical descriptors was performed using CORRELATION function in Microsoft Excel and the linear fitting program in Origin 2021. Descriptors that exhibited correlation coefficients above 0.5 with *p*-value <0.05 were considered to have statistically significant correlation with PFAS decay. When the descriptor has a strong correlation with PFAS decay, linear regression was performed.

## 3 Results and Discussion

### 3.1 Degradation of PFAS by Photolysis

**Figure 1:**
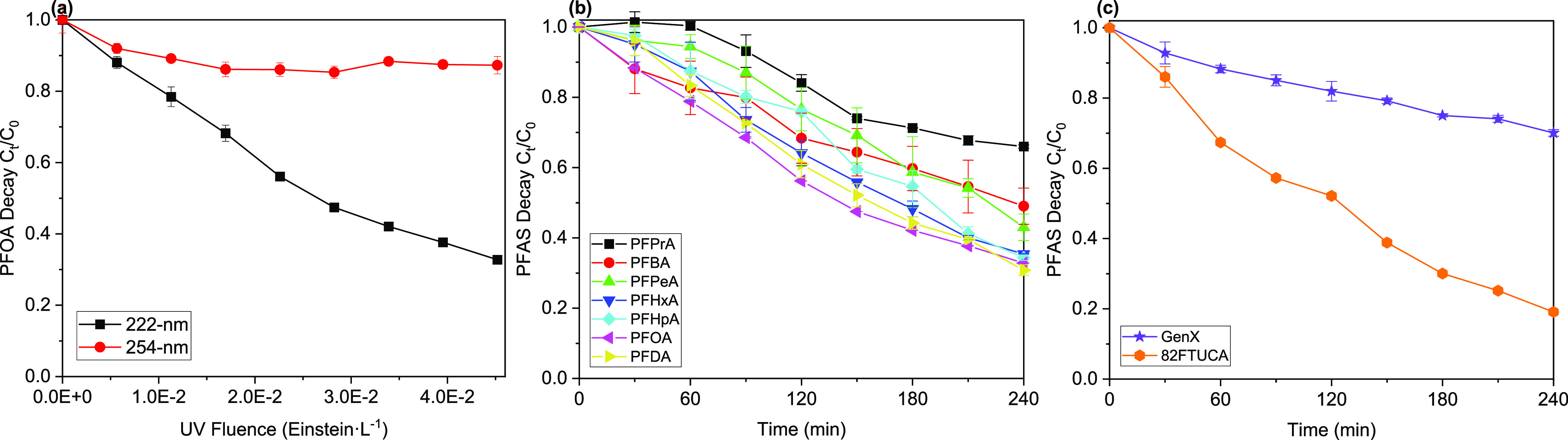
(a) Photodegradation of PFOA at different irradiation wavelengths (222 and 254 nm) but on a similar fluence basis. Reaction conditions: [PFOA]_0_ = 25.0 ± 3.7 μM, [HCO_3_^–^] = 5.0 mM, pH = 8.5. (b) Photodegradation of PFCAs (C3–C10); (c) GenX and 8:2 FTUCA under 222 nm irradiation. Reaction conditions: [PFCA]_0_ = 25.0 ± 3.2 μM, except for [PFPrA]_0_ = 44.0 ± 0.2 μM; [GenX]_0_ = 35.0 ± 1.1 μM, [8:2 FTUCA]_0_ = 11.0 ± 0.2 μM, [HCO_3_^–^] = 5.0 mM, pH = 8.5.

Potential photolysis of 19 PFAS was investigated and quantified based on the parent PFAS compound decay ([PFAS]_decay,%_ in %) and the overall defluorination (in %) at a given fluence (4.5 × 10^–2^ Einstein·L^–1^, i.e., 4.0 and 5.6 h for 222 and 254 nm irradiation, respectively). The pseudo-first-order rate constant for the decay of degradable PFAS was quantified based on time and fluence (*k* in min^–1^ and L·Einstein^–1^), determined from the slope of ln *C_t_*/*C*_0_ vs time (or fluence). Based on the concentration of fluoride ion released from the PFAS molecules into the solution, the overall defluorination ratio (deF%) was calculated by eq 1:
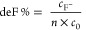
1where *c*_F^–^_ is the molar concentration of fluoride ion released in solution, *c*_0_ is the initial molar concentration of the parent PFAS, and *n* is the number of fluorine atoms in the parent PFAS molecule. Results are shown in Figure 1, Tables 2 and S3. Section 3.1 discusses PFAS parent compound degradation and defluorination, while Section 3.2 reports the quantification of degradation products and the mass balance on fluorine.

**Table 2:** Photolysis Rate Constants (Fluence-Based and Time-Based) (*k*) and Quantum Yield (Φ) of Degradable PFAS at 222 nm Irradiation

PFAS	fluence-based *k* (L·Einstein^–1^)	fluence-based *k* (cm^2^·Einstein^–1^)^a^	time-based *k* (min^–1^)	*R*^2^	quantum yield Φ (mol·Einstein^–1^)
PFPrA	9.13 ± 0.82	(9.72 ± 0.87) × 10^–3^	(1.72 ± 0.15) × 10^–3^	0.88	0.158
PFBA	15.52 ± 0.57	(16.51 ± 0.61) × 10^–3^	(2.94 ± 0.11) × 10^–3^	0.98	0.116
PFPeA	15.40 ± 0.84	(16.38 ± 0.90) × 10^–3^	(2.90 ± 0.16) × 10^–3^	0.95	0.100
PFHxA	21.71 ± 0.60	(23.10 ± 0.64) × 10^–3^	(4.09 ± 0.11) × 10^–3^	0.99	0.118
PFHpA	20.00 ± 1.02	(21.27 ± 1.08) × 10^–3^	(3.77 ± 0.19) × 10^–3^	0.96	0.102
PFOA	25.02 ± 0.73	(26.62 ± 0.78) × 10^–3^	(4.71 ± 0.14) × 10^–3^	0.98	0.137
PFOA (254 nm)	3.90 ± 0.52	(4.15 ± 0.55) × 10^–3^	(0.73 ± 0.10) × 10^–3^	0.76	--
PFDA	23.79 ± 0.63	(25.31 ± 0.67) × 10^–3^	(4.48 ± 0.12) × 10^–3^	0.99	0.104
GenX	8.19 ± 0.26	(8.71 ± 0.27) × 10^–3^	(1.54 ± 0.05) × 10^–3^	0.99	0.147
8:2 FTUCA	34.76 ± 0.76	(36.98 ± 0.81) × 10^–3^	(6.55 ± 0.14) × 10^–3^	1.00	0.031

a Fluence-based *k* (in cm^2^·Einstein^–1^) were calculated based on fluence-based *k* (in L·Einstein^–1^) and effective path length (*l* = 0.94 cm).

#### 3.1.1 PFCAs

The susceptibility of PFOA to photolysis at 222 and 254 nm irradiation was compared under the same fluence and reaction conditions (25 μM PFOA, 5 mM NaHCO_3_, pH 8.5, limited dissolved oxygen (DO)). Results showed that PFOA had significantly faster photolysis at 222 nm compared to 254 nm (*k* = (4.71 ± 0.14) × 10^–3^ > (0.73 ± 0.10) × 10^–3^min^–1^; [PFOA]_decay,%_ = 67% > 11% at 4.5 × 10^–2^ Einstein·L^–1^ fluence) (Figure 1a). Further, significant degradation and defluorination were observed for all PFCAs (C_*n*_F_2*n*+1_COO^–^, *n* = 3–10) at 222 nm, although much slower decay was found with PFPrA (*n* = 3) ([PFAS]_decay,%_ = 34% for PFPrA, compared to 51–69% for *n* ≥ 4 PFCAs). The *k* value of PFCAs’ degradation increased with the carbon number, from (1.72 ± 0.15) × 10^–3^ min^–1^ for PFPrA to (4.71 ± 0.14) × 10^–3^ min^–1^ for PFOA, indicating the impact of chain length.

#### 3.1.2 GenX, 8:2 FTUCA, and Other PFAS

GenX and 8:2 FTUCA were also found to degrade under 222 nm irradiation ([PFAS]_decay,%_ = 30% for GenX and 81% for 8:2 FTUCA at 4.5 × 10^–2^ Einstein·L^–1^ fluence, Table S3). GenX contains an ether linkage, while 8:2 FTUCA contains an unsaturated C=C bond (Table S1). Compared to PFCAs of the same carbon number, the photolysis rate of GenX was slower (*k*_Genx_ = (1.54 ± 0.05) × 10^–3^ min^–1^) than PFHxA, whereas the photolysis rate of 8:2 FTUCA was faster (*k*_8:2FTUCA_ = (6.55 ± 0.14) × 10^–3^ min^–1^) than PFDA (Figure 1b–c and Table 2). Other PFAS, including perfluorosulfonates (C5-C8 PFSAs), fluorotelomer phosphate diester (6:2 diPAP), fluorotelomer carboxylic acids (5:3 FTCA and 6:2 FTS), and perfluorosulfonamides (FOSA and FHxSA), showed minimal degradation under 222 nm irradiation. Overall, the photolysis rate constants of PFAS under 222 nm irradiation follow the order of 8:2 FTUCA > PFCAs > GenX > > others (Table 2).

#### 3.1.3 Impact of Reaction Conditions

The impacts of varying reaction conditions on direct photolysis under 222 nm irradiation were evaluated for PFOA (Figure S4, Tables S3 and S4). Results showed that varied pH from 5.0 to 10.5, or the presence or absence of DO, had a minimal impact on the photolysis rate constant and decay % of PFOA (Figure S4(a,b)).

The effects of HCO_3_^–^, HPO_4_^2–^/H_2_PO_4_^–^, and Cl^–^ anions were investigated, in comparison to a clean water matrix (MQW) (Figure S4(c)). Unlike the scavenging effect of bicarbonate/carbonate on advanced oxidation processes (AOPs),^48^ the presence of HCO_3_^–^ had a minimal impact on PFOA photolysis rate ([PFOA]_decay,%_ = 67% for HCO_3_^–^, compared to 65% for MQW; *k* = (4.71 ± 0.14) × 10^–3^ for HCO_3_^–^, compared to (4.23 ± 0.13) × 10^–3^ for MQW). Phosphate ions also had a negligible effect, while the presence of Cl^–^ slightly increased the degradation extent and rate ([PFOA]_decay,%_ increased from 65 to 74%; *k* increased from (4.23 ± 0.13) × 10^–3^ to (5.18 ± 0.14) × 10^–3^ min^–1^).

Further, photolysis of PFOA was evaluated in a real wastewater sample (Text S2). The wastewater characteristics were: pH = 7.0, [NO_3_^–^] = 11 mg·L^–1^, [NO_2_^–^] = 0.02 mg·L^–1^, [total-P] = 0.03 mg/L, and [total organic carbon (TOC)] = 6 mg/L. In comparison to the bicarbonate buffer, the rate constant and extent of PFOA degradation were reduced significantly in the wastewater ([PFOA]_decay,%_ decreased from 67 to 25%; *k* decreased from (4.71 ± 0.14) × 10^–3^ to (0.94 ± 0.07) × 10^–3^ min^–1^; Figure S4(d)). Common constituents, such as nitrate and dissolved organic matter (DOM), in real water matrices could influence photolysis of PFAS by different mechanisms, such as light-shielding, scavenging, and catalysis effects.^49−52^

Humic acids (HAs) were selected as model natural organic matter (NOM), since HAs make up about 70% of NOM.^53^ The presence of HA (10 mg·L^–1^) slightly increased the degradation extent and rate of PFOA ([PFOA]_decay,%_ increased from 67 to 71%; *k* increased from (4.71 ± 0.14) × 10^–3^ to (5.17 ± 0.09) × 10^–3^ min^–1^) (Tables S3 and S4), which indicated that DOM in the real water matrix was not a contributor to the significant decrease in PFOA degradation.

Nitrate (NO_3_^–^) and nitrite (NO_2_^–^) both absorb light strongly in the 200–250 nm region with high molar absorption coefficients (ε = 2500 and 3500 M^–1^ cm^–1^ at 222 nm, respectively).^54^ As NO_3_^–^ is commonly detected and typically much more abundant than NO_2_^–^ in wastewater effluent, we evaluated the effect of NO_3_^–^ at 0.5–15 mg·L^–1^. *k* and [PFOA]_decay,%_ decreased with the increase of NO_3_^–^ concentration (Figure S4(e)). Furthermore, the clean water spiked with 10–15 mg·L^–1^ nitrate and the real wastewater sample ([NO_3_^–^] = 11 mg·L^–1^) exhibited similar PFOA photolysis degradation extent and rate ([PFOA]_decay,%_ = 21–24% for 10–15 mg·L^–1^ NO_3_^–^, 25% for real wastewater; *k* = (0.78 ± 0.06) × 10^–3^ – (1.17 ± 0.10) × 10^–3^ min^–1^ for 10–15 mg·L^–1^ NO_3_^–^, (0.94 ± 0.07) × 10^–3^ for real wastewater). These results suggested that nitrate in the real water matrix was the dominant factor that hindered the photolysis of PFOA under 222 nm irradiation, probably by light shielding as the light transmittance is expected to decrease from 95 to 25% when nitrate concentration was increased from 0.5 to 15 mg·L^–1^. Although UV/NO_3_^–^ system can generate hydroxyl radical (^•^OH) as well as reactive nitrogen species (RNS) such as nitric oxide (^•^NO), nitrogen dioxide (^•^NO_2_), and peroxynitrite (ONOO^–^),^55,56^ previous studies have shown that AOPs based on ^•^OH are ineffective in the treatment of PFAS.^32,57^ Overall, PFOA could undergo direct photolysis under 222 nm irradiation with the presence of a moderate concentration of NO_3_^–^.

### 3.2 Generation of Degradation Products

#### 3.2.1 Degradation Products of PFAS

**Figure 2:**
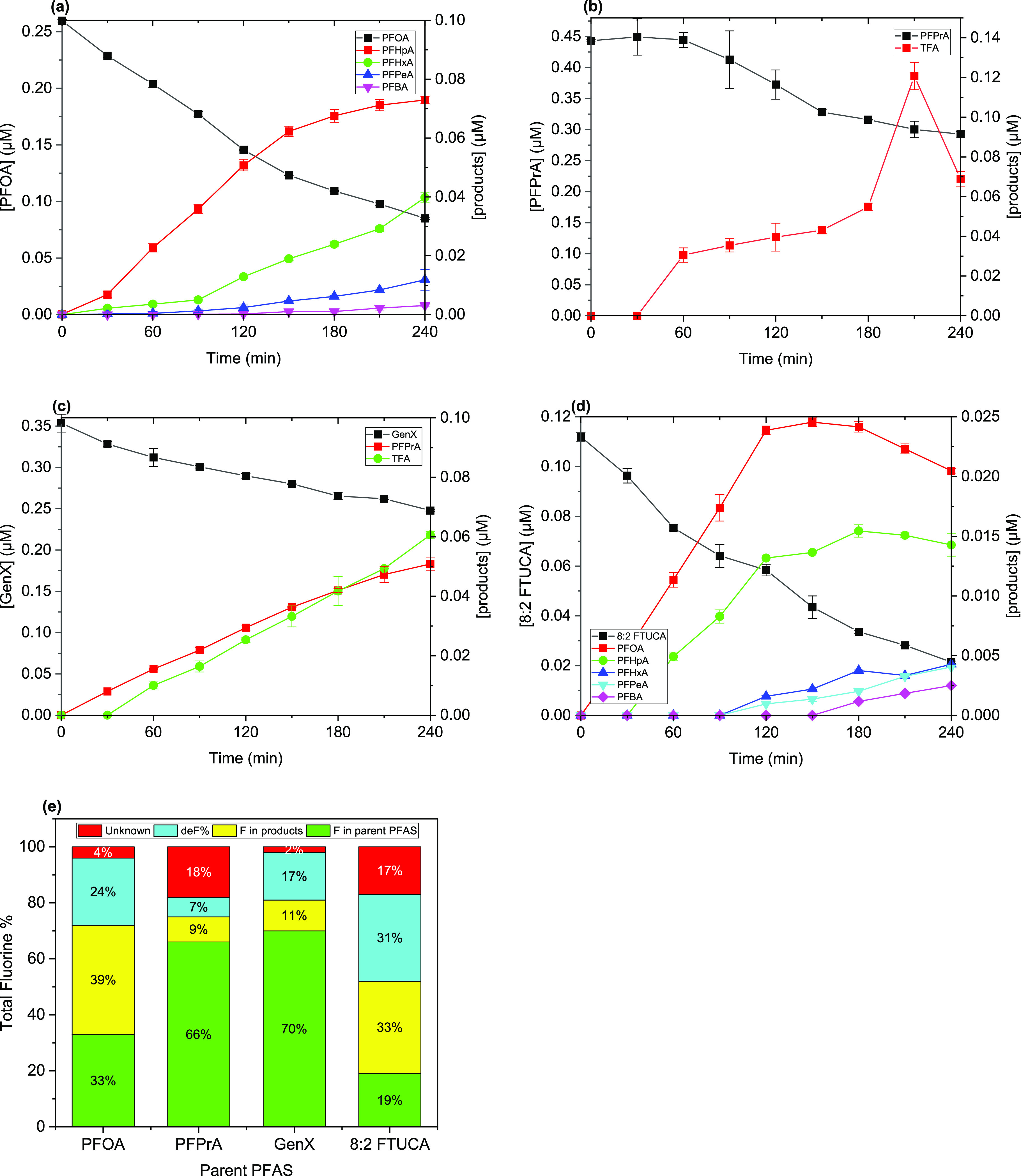
Representative degradation products of degradable PFAS: (a) PFOA; (b) PFPrA; (c) GenX; (d) 8:2 FTUCA; and (e) mass balance of fluorine (F). Reaction condition: [PFOA]_0_ = 25.0 ± 1.2 μM, [PFPrA]_0_ = 44.0 ± 0.18 μM, [GenX]_0_ = 35.0 ± 1.1 μM, [8:2 FTUCA]_0_ = 11.0 ± 0.2 μM, [HCO_3_^–^] = 5 mM, initial pH = 8.5.

The generation of degradation products, deF% and the mass balance of fluorine for four (PFOA, PFPrA, GenX, and 8:2 FTUCA) of the degradable PFAS are shown in Figure 2, while the entire results are reported in Table S5.

The photolysis of PFCAs at 222 nm produced shorter-chain PFCAs as their major degradation products (Figure 2a,b,e and Table S5). For example, PFOA generated four shorter-chain PFCAs (*C* ≤ 7): PFHpA, PFHxA, PFPeA, and PFBA. PFPrA produced TFA (*C* = 2). The mass balance of fluorine consisted of organic fluorine (in detected products), F^–^ ion and unidentified fluorine. The fraction of unidentified products was small (4–18%), and thus it could be inferred that short-chain PFCA and F^–^ were the main products formed by direct photolysis of PFCAs. The dominant formation of shorter-chain PFCA and F^–^ also indicated that the degradation likely involved cleavage of the C–C bond between the fluoroalkyl chain and carboxyl group (i.e., decarboxylation), as well as cleavage of the C–F bonds. Insights into the plausible mechanisms of PFCA photolysis are presented in Section 3.3.

GenX produced TFA and PFPrA at 1:1 molar ratio, and the fraction of unidentified products was only 2% (Figure 2c,e and Table S5). These results suggest that the GenX molecule was split at the ether group (−O−) and formed PFPrA and TFA under 222 nm irradiation, which is similar to that proposed in the UV_254_/sulfite system.^58^

Photolysis of 8:2 FTUCA also generated shorter-chain PFCAs (*C* ≤ 8) as major degradation products (Figure 2d,e and SI Table S5). The fraction of unidentified products was 17%. Although the mechanism of FTUCA’s defluorination by photodegradation is not fully clear, the generation of PFOA as the main product strongly suggests that UV-222 irradiation of 8:2 FTUCA may first cleave the C=C bond between C_8_F_16_ and CHCOOH, and the resulted C_8_F_16_ radical could then transform to PFOA (C_7_F_15_COOH).

#### 3.2.2 Impacts of Reaction Conditions on Products Generation

As discussed earlier, solution pH, DO, and anions (bicarbonate, phosphate and chloride) had negligible to minor effects on parent PFOA degradation/defluorination under 222 nm irradiation. Change of solution pH (5.0 to 10.5) also did not affect the degradation products and mass balance of fluorine. The fraction of unidentified products of all reactions conducted in bicarbonate buffer were limited to 2–10% for PFOA, and 2–18% for other degradable PFAS. However, the presence of DO, certain anions (HPO_4_^2–^/H_2_PO_4_^–^, Cl^–^, and NO_3_^–^), and HA showed significant impacts on the product generation from PFOA. Generally, they reduced the formation of shorter-chain PFCAs while increased the fraction of unidentified products (Table S5 and Figures S5–S7). The mass balance of fluorine at the end of reaction showed that the fraction of the unidentified products increased from 4 to 24%, 36%, 24%, 38%, and 17% in the presence of DO, HPO_4_^2–^/H_2_PO_4_^–^, Cl^–^, HA, and NO_3_^–^, respectively. These results suggest that under 222 nm irradiation, DO, HPO_4_^2–^/H_2_PO_4_^–^, Cl^–^, HA, and NO_3_^–^ may generate reactive species and/or interact with intermediates of PFAS photolysis to produce additional unknown products. Note that the increase of NO_3_^–^ concentrations (0.5–15 mg·L^–1^) did not significantly change the fraction of unidentified products (14–17%, Table S5). More study is needed to elucidate these effects in depth.

### 3.3 Mechanistic Insights for PFAS Degradation at 222 nm

#### 3.3.1 PFAS Absorptivity and Quantum Yield at 222 nm

The experimental results indicate that direct photolysis is the dominant pathway for the decay and defluorination of PFAS under 222 nm irradiation. It is well known that major factors affecting the direct photolysis of organic molecules include the molar absorption coefficient and quantum yield. The molar absorption coefficients of the 19 PFAS at 222 nm (ε_222_ in M^–1^·cm^–1^) ranged at 27–106 for PFCAs, 26 for GenX, 520 for 8:2 FTUCA, 14–47 for PFSAs, 1319 for 6:2 diPAP, 544 for 6:2 FTCA, 799 for 5:3 FTCA, 35 for 6:2 FTS, 86 for FOSA, and 18 for FHxSA (Table S6).

The quantum yield (Φ) of degradable PFAS can be calculated using eq 2:^59,60^
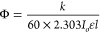
2where Φ is the quantum yield (in mol·Einstein^–1^); *k* is the rate constant of PFAS degradation under 222 nm irradiation (in min^–1^); *I*_o_ is the fluence rate of the incident light at 222 nm (3.14 × 10^–6^ Einstein·L^–1^·s^–1^); ε is the molar absorption coefficient of PFAS (in M^–1^·cm^–1^) at 222 nm; and *l* is the effective path length of reactor (0.94 cm in this study). Note that the PFAS photolysis was in dilute solution in this study with the fraction of light absorption by the system far less than 0.1, and eq 2 is suitable to determine the quantum yield.^61^ The derived quantum yields of PFCAs, GenX, and 8:2 FTUCA at 222 nm ranged from 0.031 to 0.158 (Table 2).

**Figure 3:**
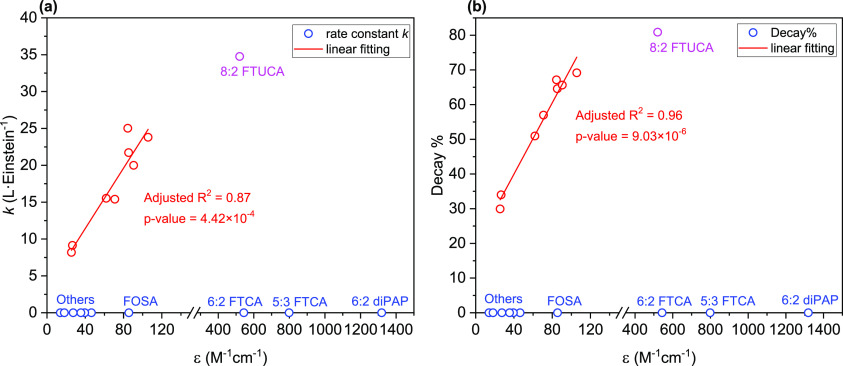
Correlation of PFAS molar absorption coefficients at 222 nm wavelength with (a) fluence-based rate constants (*k*); (b) overall decay% of PFAS. Red circles are data of PFCAs (C3–C10) and GenX, purple circle is data of 8:2 FTUCA, blue circles are data of 6:2 diPAP, FTCAs, FOSA and others (PFSAs (C5–C8), 6:2 FTS, and FHxSA).

No obvious correlation between the quantum yield and the rate and extent of PFAS degradation under 222 nm irradiation could be found. On the other hand, molar absorption coefficient had a strong impact. Analysis indicated statistically significant linear correlations of the molar absorption coefficient with the direct photolysis rate constant and the overall decay % of PFCAs and GenX (*p* < 0.05) (Figure 3 and Table S7)—a higher molar absorption coefficient led to a higher rate constant and extent of degradation under 222 nm irradiation. However, molar absorption coefficient alone cannot explain the differences among all the 19 PFAS. Although 8:2 FTUCA has a considerably higher ε_222_ value than PFCAs and GenX, its direct photolysis *k* and decay% were not proportionally greater than PFCAs and GenX. Also, the high ε_222_ values of FTCAs and 6:2 diPAP did not make contribution to their degradation under 222 nm irradiation. These results indicated that the light absorptivity of PFAS is not the only factor determining the susceptibility toward direct photolysis.

#### 3.3.2 Relevance of Decarboxylation and DFT Calculations

Among the PFAS, the compounds that readily undergo photolysis at 222 nm are PFCAs, 8:2 FTUCA and GenX, which points to the importance of the carboxylate group. While 6:2 FTCA and 5:3 FTCA also contain a carboxylate end group, they differ from 8:2 FTUCA in a non-activating (C–C) versus an activating (C=C) moiety adjacent to the carboxylate group (Table S1).

Previous research proposed eq 3–6 as a possible mechanism for the formation of short-chain PFCAs from direct photolysis of PFOA at 185 and 220–460 nm range:^32,34^
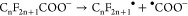
3
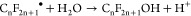
4
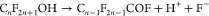
5
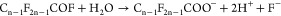
6Briefly, UV irradiation of a longer-chain PFCA cleaves the C–C bond between C_n_F_2n+1_ and the carboxylate group, forming C_n_F_2n+1_ radical (eq 3). The C_n_F_2n+1_ radical generates a hydrated product, C_n_F_2n+1_OH, in water (eq 4), followed by HF elimination to form C_n–1_F_2n–1_COF (eq 5). Finally, hydrolysis of C_n–1_F_2n–1_COF generates C_n–1_F_2n–1_COO^–^ (eq 6).

Eq 3 is a decarboxylation reaction crucial for the degradation to occur. Therefore, the bond dissociation energy (BDE) for the αC–C bond between the fluoroalkyl chain and carboxyl group was calculated with DFT to see if they could be used to explain the observed trends in reactivity, as a lower BDE implies that the bond is more easily cleaved. These calculations showed that the αC–C BDEs for longer-chain PFCAs (*C* ≥ 6) (82.63–84.54 kcal·mol^–1^) are lower than those for the shorter-chain PFCAs (*C* < 5) (85.03–86.70 kcal·mol^–1^) (Table S8). This trend agreed with the results that longer-chain PFCAs degrade faster than the shorter-chain ones (e.g., PFPrA (*k* = 9.13 L·Einstein^–1^) compared to longer-chain ones such as PFOA (*k* = 25.02 L·Einstein^–1^) and PFDA (*k* = 23.79 L·Einstein^–1^)). Statistical analysis indicated that there were weak correlations of the αC–C BDEs with the direct photolysis rate constant (*p* = 0.031) and the overall decay % (*p* = 0.054) of PFCAs (Figure S8(a,b)). It is worth noting that the photon energy of 222 nm (539 kJ·Einstein^–1^ or 129 kcal·Einstein^–1^) is greater than these BDEs.

It is known that the decarboxylation tendency of carboxyl-containing compounds decreases as p*K*_a_ of the carboxylic acid group decreases (i.e., increased acidity, due to the neighboring inductive effect of electron-withdrawing groups, for example).^62,63^ While the reported p*K*_a_ values of PFCAs have wide ranges, PFPrA generally has a considerably lower p*K*_a_ compared to long-chain PFCAs (0.5 for PFPrA vs 2.3 for PFOA).^64,65^ This trend agreed with the results that the degradation rate and extent of PFPrA decomposition were lower than the longer-chain PFCAs.

The chain length of PFAS may also affect molecular conformation. Unlike PFPrA, PFCAs with a chain length greater than C4 can exhibit a stable helical conformation, characterized by a torsional twist in the central F–C–C–F or C–C–C–C dihedral angle.^66^ To minimize the complexities of performing conformational sampling, only the straight-chain conformers were considered in this study; however, the impact of conformational isomerism on the calculated properties reported here is currently underway and will be reported in a future study. Another chain length effect is the “1,2-F atom rearrangement” that could occur in perfluoroalkyl radicals resulting from cleavage of the carboxyl group. Previous theoretical calculations indicated that fluorine atoms can migrate to a neighboring carbon accompanying the relocation of unpaired electrons to create branched carbon radicals (example illustration in Figure S9), and such rearrangement helps increase the stability of perfluoroalkyl radicals.^67^ Van Hoomissen and Vyas calculated the Δ*G* for 1,2-F atom rearrangements and found that PFPrA had a relatively greater Δ*G* compared to PFAS with more than 4 carbons.^67^ This implies that the 1,2-F atom rearrangement in the PFPrA molecule is less favorable than in longer-chain PFCAs, decreasing the stability of its perfluoroalkyl radical.

As defluorination involves cleavage of C–F bonds, the BDE of the αC–F bond between the α-carbon and fluorine atoms might be important according to the proposed eq 5 and thus was calculated. However, the αC–F BDEs for PFCAs were limited to a very narrow range of 110.50–111.99 kcal·mol^–1^ (Table S8) and no significant trend could be observed (*p* > 0.05) (Figure S8(c,d)).

For GenX, 8:2 FTUCA, and PFOS (Table 1), the BDEs of the C-O bonds in GenX (61.15 and 79.80 kcal·mol^–1^) were lower compared to the αC–C BDE of PFHxA with the same carbon number (84.54 kcal·mol^–1^). However, lower BDE did not contribute to faster GenX degradation under 222 nm irradiation. On the other hand, the BDEs of C–C and C=C in the 8:2 FTUCA structure (97.26, 170.39 kcal·mol^–1^ respectively) were higher compared to the αC–C BDE of PFDA with the same carbon number (82.63 kcal·mol^–1^), while the degradation rate and extent of 8:2 FTUCA were faster than those of PFDA. The low BDE of C–S bond in PFOS structure compared to the αC–C BDEs in PFCAs did not lead to more favorable degradation at all. Therefore, the BDEs of individual bonds in these PFAS structures cannot be used to explain their photolysis reactivity under 222 nm irradiation.

In the proposed mechanism mentioned earlier, there were two steps that were likely candidates for being rate-determining; the initial photolytic cleavage of the αC–C bond and the formation of the acyl fluoride. Completely modeling the photochemical pathway is nontrivial and beyond the scope of the current work; however, it will be the focus of a future study. Estimating the barrier heights for the thermal formation of acyl fluorides was performed by calculating the Gibbs free energy of activation (Δ*G*^‡^) for a series of PFCAs. The proposed intermediates of C_n_F_2n+1_OH and C_n–1_F_2n–1_COF have not been confirmed experimentally in the literature. Here, we calculated the activation energies for the HF elimination step of eq 5 for the PFCAs, and the calculated values are summarized in Table S10. Intriguingly, the results showed that Δ*G*^‡^ of the HF elimination step for PFCAs increased as the number of carbons in the structure was decreased, consistent with the observed trend in reactivity. Despite this inverse linear correlation between Δ*G*^‡^ and the degradation rate constant *k* and overall decay % of PFCAs (*p* < 0.05) (Table S11 and Figure S8(e,f)), however, the calculated Δ*G*^‡^ values for all of the PFCAs (*C* = 3–10) were very large (37.32–40.85 kcal·mol^–1^). Such large activation barriers indicates that the proposed HF elimination step (eq 5) is unlikely to proceed at room temperature. The calculated Δ*G*^‡^ value in this study is lower than the value calculated by M06-2X-D3(0)/def2-TZVPPD in a previous study (38.51 compared to 48.16 kcal·mol^–1^ for PFOA); however, it is still very large and so the same conclusion is reached, which is that this reaction step has too high of an activation energy to be a primary mechanism.^68^ These results suggest that part of the widely proposed PFCA degradation pathway is likely inaccurate, and thus photolysis of PFCAs at 222 nm does not involve the elimination of HF from alcohol to form an acyl fluoride (converting C_n_F_2n+1_OH to C_n–1_F_2n–1_COF). Further mechanistic investigation on the exact degradation pathways is thus warranted and should be pursued in future research.

## 4 Conclusions

Overall, this study demonstrates that PFCAs are much more susceptible to direct photolysis by UV irradiation at 222 nm than at 254 nm, and the degradation involves decarboxylation and defluorination (cleavage of C–C bond and C–F bond) to form shorter-chain PFCAs and free fluoride. GenX and FTUCAs are also susceptible to direct photolysis at 222 nm irradiation, which follows more complicated degradation mechanism different from that of PFCAs and requires further elucidation. Other PFAS, including PFSAs, 6:2 diPAP, 5:3 FTCA, 6:2 FTS, FOSA, and FHxSA, do not undergo photolysis under 222 nm irradiation. The different photolysis rates among PFCAs of various carbon-chain length showed a strong dependence on their molar absorption coefficients, but may also be influenced by other chain-length-dependent structural factors that affect the carboxylic acid p*K*_a_, perfluoroalkyl chain conformation, and stability of perfluoroalkyl radicals. As a theoretical reference for PFAS degradation pathways and mechanisms, computational results revealed that (1) BDEs of specific bonds in the PFAS structures cannot explain the different photolysis rates of degradable PFAS, or the resistance of the other PFAS to direct photolysis at 222 nm irradiation; (2) HF elimination from the alcohol (C_n_F_2n+1_OH), which was commonly proposed in earlier studies as an intermediate during PFCA degradation, is an unlikely pathway due to high activation barriers. Future research should prioritize elucidating the specific pathways of PFAS degradation after decarboxylation induced by 222 nm irradiation.

Results of this study suggest that the photolysis rate of degradable PFAS at 222 nm irradiation were not significantly influenced by the aqueous solution matrix except for the presence of strong light-absorbing species such as nitrate. The far-UVC technologies are worth further exploring as 222 nm irradiation is not only safer for human tissues, but also can achieve significant decomposition of some PFAS (PFCAs, GenX, and FTUCAs) in aqueous solutions without addition of chemicals. Future research should further investigate the performance of the KrCl* excimer lamps under practical conditions for a broader range of real water matrices, PFAS compounds and co-contaminants, and the application of far-UVC with reductants and/or oxidants for enhanced advanced reduction and/or oxidation.

## Supporting Information Available

Chemicals and reaction solution preparation; experimental setup; analytical method; theoretical calculations; information on selected PFAS; PFAS analytes, theoretical m/z, and retention time; PFAS decay and defluorination; fluence-based and time-based rate constants (*k*) of PFOA; products and mass balance of fluorine; UV absorbance at 222 nm wavelength of PFAS solutions and corresponding molar absorption coefficients (ε); statistical analysis of correlations; calculated BDEs (kcal·mol^–1^); calculated activation energies (Δ*G*^‡^, kcal·mol^–1^); Cartesian coordinates of optimized PFAS molecules; illustration of experimental setup; effect of different reaction conditions on the photodegradation of PFOA; representative degradation products of degradable PFOA; correlation of activation energies for PFCAs with PFAS decay; and scheme of 1,2-F atom rearrangements in perfluoropropyl, perfluorobutyl, and perfluoropentyl radical systems (PDF)
