# Supplementary material for: Degradation and Defluorination of Per- and Polyfluoroalkyl Substances by Direct Photolysis at 222 nm
Source: ACS ES T Water. 2023 Jul 6;3(8):2776–85. doi: 10.1021/acsestwater.3c00274 (PMC10425954; doi:10.1021/acsestwater.3c00274)
Supplement: Supplementary file 1 — ew3c00274_si_001.pdf [file ew3c00274_si_001.pdf]

Supporting Information

for

Degradation and Defluorination of Per- and Polyfluoroalkyl Substances (PFAS) by Direct Photolysis at 222 nm

Xiaoyue Xin,[a] Juhee Kim,[a] Daniel C. Ashley,[b] and Ching-Hua Huang[a,*]

[a]School of Civil and Environmental Engineering, Georgia Institute of Technology, Atlanta, GA 30332, United States

[b]Department of Chemistry and Biochemistry, Spelman College, Atlanta, GA 30314, United States

[*]Corresponding Authors. Emails: ching-hua.huang@ce.gatech.edu (Ching-Hua Huang)

Number of Pages: 48

Number of Texts: 4

Number of Tables: 12

Number of Figures: 9

Number of References: 15

Contents

Texts

Text S1. Chemicals and reaction solution preparation. ---S4

Text S2. Experimental setup. ---S5

Text S3. Analytical method. --S5

Text S4. Theoretical calculations. ---S6

Tables

Table S1. Information of 19 selected PFAS in this study. --S7

Table S2. PFAS analytes, theoretical m/z, and retention time. --S9

Table S3. Experimental results for PFAS decay and defluorination under 222 nm. --------------------S10

Table S4. Fluence-based and time-based rate constants (*k*) of PFOA under 222-nm irradiation and various reaction conditions. ---S12

Table S5. Results of quantification of products and mass balance of fluorine (F) for PFAS degradation. ---S13

Table S6. UV absorbance at 222-nm wavelength of PFAS stock aqueous solutions and corresponding molar absorption coefficients (ε). ---S15

Table S7. Statistical analysis of correlations between the degradation rate constants (*k*), overall decay of PFCAs and GenX and their molar absorption coefficients (ε) at the 222 nm wavelength. ---S16

Table S8. Calculated αC-C and αC-F BDEs (kcal·mol^{-1}) for PFCAs. ----------------------------------S16

Table S9. Calculated BDEs (kcal·mol^{-1}) for PFOS, GenX and 8:2 FTUCA. --------------------------S17

Table S10. Calculated activation energies (Δ G‡, kcal·mol^{-1}) for PFCAs. ----------------------------- S18

Table S11. Statistical analysis of correlations between the degradation rate constants (*k*), overall decay% of PFCAs and their activation energies (Δ G‡) for the HF elimination step. -----S18

Table S12. Cartesian coordinates of optimized PFAS molecules.--------------------------------------S26

Figures

Figure S1. Illustration of experimental set-up using 222-nm far UV-C lamp. ------------------------S19

Figure S2. Illustration of experimental set-up using 254-nm LP UV lamp. ----------------------------S19

Figure S3. The adsorption of F^- on the quartz reactor: change in concentration for 4 h irradiation at 222 nm. --S20

Figure S4. Effect of different reaction conditions on the photodegradation of PFOA under 222-nm irradiation. ---S21

Figure S5. Representative degradation products of degradable PFOA with the presence of dissolved oxygen. --S22

Figure S6. Representative degradation products of degradable PFOA with the presence of different anions. ---S23

Figure S7. Representative degradation products of degradable PFOA. ---------------------------------S24

Figure S8. Correlation of activation energies for PFCAs (C3-C10) with (a) the fluence-based rate constants (*k*); (b) overall decay% of PFAS. ---S25

Figure S9. Scheme of 1,2-F Atom Rearrangements in Perfluoropropyl (1A), Perfluorobutyl (1B, 2B), and Perfluoropentyl (1C–5C) Radical Systems. ---S26

Text S1. Chemicals and Solution Preparation.

The name and structure information of selected 19 PFAS in this study is summarized in **Table S1**. All PFAS chemicals were purchased from Toronto Research Chemicals and Sigma-Aldrich with the highest purity. Most individual PFAS compounds were dissolved in Milli-Q water to make 1.0 mM stock solutions. For 6:2 diPAP, methanol was added to facilitate the dissolution, giving 0.3% (v/v) methanol in the 20 mL reaction solution. Methanol does not interfere with the PFAS decay and defluorination by direct photolysis. All PFAS stock solutions were stored in polypropylene (PP) tubes at 5°C.

Sodium bicarbonate (≥99.7%; Sigma-Aldrich), sodium phosphate monobasic (≥99.0%; Sigma-Aldrich), sodium phosphate dibasic (≥99.0%; Sigma-Aldrich), sodium chloride (≥99.0%; Sigma-Aldrich), sodium nitrate (≥99.0%; Sigma-Aldrich), humic acid (≥90%; MP Biochemicals), sodium hydroxide (97%; Thermo Fisher Scientific), and hydrochloric acid (35%, Thermo Fisher Scientific) were used to prepare photolysis reaction solutions. LC-MS grade methanol (Fisher Optima), LC-MS grade acetonitrile (Fisher Optima), acetic acid (Fisher Optima), and LC-MS grade ammonium acetate (Fisher Optima) were used to prepare LC eluents. Reagent-grade deionized (DI) water (>18mΩ-cm) was generated from a Milli-Q Nanopure water purification system (Billerica, MA).

The PFAS standards for LC-TOFMS analysis were purchased from Wellington Laboratories (Guelph, Canada), including perfluorobutanoic acid (PFBA), perfluoropentanoic acid (PFPeA), perfluorohexanoic acid (PFHxA), perfluoroheptanoic acid (PFHpA), perfluorooctanoic acid (PFOA), perfluorononanoic acid (PFNA), perfluorodecanoic acid (PFDA), perfluorobutane sulfonic acid (PFBS), perfluoropentane sulfonic acid (PFPS), perfluorohexane sulfonic acid (PFHxS), perfluorheptane sulfonic acid (PFHpS), perfluorooctane sulfonic acid (PFOS), 6:2 fluorotelomer sulfonic acid (6:2 FTS), 6:2 fluorotelomer carboxylic acid (6:2 FTCA), 5:3 fluorotelomer carboxylic acid (5:3 FTCA), perfluorohexane sulfonamide (FHxSA), perfluorooctane sulfonamide (PFOSA), hexafluoropropylene oxide-dimer acid (HFPO-DA or GenX), 6:2 fluorotelomer phosphate diester (6:2 diPAP), isotope-labeled 6:2 fluorotelomer phosphate diester ([$^{13}C_2$] 6:2 diPAP), and 8:2 fluorotelomer unsaturated carboxylic acid (8:2 FTUCA). Trifluoroacetic acid (TFA) was purchased from Agilent (Santa Clara, CA).

Text S2. Experimental Setup.

Comparison of direct photolysis under 222-nm and 254-nm irradiation. The experiments under 222-nm irradiation were conducted in a sealed quartz reactor with 20 mL solution containing around 25 μM PFOA (except for PFPrA (44 ± 0.18 μM), GenX (35 ± 1.08 μM) and 8:2 FTUCA (11 ± 0.17 μM)), and 5 mM $NaHCO_3$ (pH 8.5), as described in the main text and illustrated in **Figure S1**. Iodide–iodate actinometry was applied to measure the UV fluence rate received in the reaction solution, which has been proven to be accurate for both 222-nm and 254-nm irradiation.[1,2] The UV fluence rate received in the reaction solution was measured to be 3.14×10^{-6} Einstein·L^{-1}·s^{-1}. The effective path length is 0.94 cm. The solutions were initially purged by nitrogen gas for 0.5 h to remove dissolved oxygen.

The experiments under 254-nm irradiation were conducted in a cylindrical quartz reactor with a quartz plate on the top in a chamber equipped with a low pressure UV lamp (G4T5 Hg lamp, Philips TUV4W) (illustrated in **Figure S2**). Continuous magnetic stirring was provided during experiments to guarantee the reactor was well mixed under room temperature. The UV fluence rate was determined to be

2.23×10^{-6} Einstein·L^{-1}·s^{-1} using iodide–iodate actinometry.[1] The reaction solution (20 mL) was prepared the same as reactions under 222-nm irradiation. To ensure the same fluence was received by the reaction solutions, the experiments under 222-nm irradiation were conducted for 4 h, and the experiments under 254-nm irradiation were conducted for 5.6 h.

Impact of varying reaction conditions.

Effect of solution pH. The reaction solutions containing 5 mM $NaHCO_3$ were adjusted by NaOH and HCl to the range of desired pHs (5.0, 6.0, 7.0, 8.5 and 10.5).

Effect of dissolved oxygen (DO). To evaluate the impact of presence of DO on PFOA degradation, experiments were conducted in the same quartz reactor and under the same condition as described above (20 mL solution containing 25 µM PFOA, 5 mM $NaHCO_3$ (pH 8.5)), except that the plate on the quartz reactor was removed, and reaction solutions were not purged with N_2 gas before reaction.

Effect of solution anions. Solutions containing 5 mM $NaHCO_3$ (pH = 8.5), 5 mM Na_2HPO_4/NaH_2PO_4, (pH = 7.0), 5 mM NaCl (pH = 7.0) and Milli-Q water (no pH adjustment) were used as reaction solutions for PFOA degradation. Solution pH was adjusted by small amounts of NaOH and HCl. All reactions were carried out in the sealed quartz reactor with 20 mL solution containing around 25 µM PFOA under 222-nm irradiation as described above. Solutions were initially purged by nitrogen gas for 0.5 h.

Effect of real water matrix. Wastewater samples after a ultrafiltration (UF) treatment process were collected from a local municipal wastewater treatment plant. PFOA was spiked into the wastewater and then the sample was evaluated for PFOA degradation. The concentrations of NO_2^- and NO_3^- in the real wastewater samples were determined using HACH HR NitriVer 3 and NitraVer 5 (Hach Company, Loveland, CO) powder pillows with a spectrophotometer at 548 nm and 392 nm, respectively. Solutions containing 10 ppm humic acid (HA) and 5 mM $NaHCO_3$ (pH = 7.0) were used as reaction solutions for PFOA degradation with the presence of DOM. All reactions were carried out in the sealed quartz reactor with 20 mL solution containing around 35 µM PFOA under 222-nm irradiation as described above. The solutions were initially purged by nitrogen gas for 0.5 h.

Effect of nitrate. Solutions containing different concentration of $NaNO_3$ (0.5, 5, 10, 15 ppm) were prepared to evaluate the effect of nitrate. All reactions were carried out in the sealed quartz reactor with 20 mL solution containing around 35 µM PFOA under 222-nm irradiation as described above. Solutions were initially purged by nitrogen gas for 0.5 h, and solution pH was adjusted to 7.5 by NaOH and HCl.

Text S3. Analytical Method.

LC-TOFMS Analysis. Samples were injected to an Agilent 1260 Infinity high performance liquid chromatography (HPLC) with 6230 time of flight mass spectrometry (TOFMS) system under electrospray ionization in negative (ESI^-) ion mode. Full scan mass spectra were collected from 50 to 1000 m/z with a mass error of < 10 ppm. The drying gas flow rate was 6 L·min^{-1}, gas temperature 200 ^oC, nebulizer pressure 40 psi, capillary voltage 4000 V, and fragmentation voltage 140 V. Reference standards with masses of 119.036320 and 980.016375 were used to continually correct mass accuracy. Sample injection volumes were 20.0 µL. Two different columns were used: a Poroshell 120 EC–C18

column (2.1×150 mm, 4 μm) for long-chain PFAS and a RSpak JJ-50 2D column (2.0×150 mm, 5 μm) for short-chain PFAS. The chromatographic method for Poroshell 120 EC–C18 column consisted of a multi-step gradient lasting 23.5 min at a constant 0.3 $mL\cdot min^{-1}$ eluent flow rate with 5.0 mM ammonium acetate and 80/20 (v/v) methanol/acetonitrile. After 2 min hold at 100% of 5.0 mM ammonium acetate, the organic phase was ramped to 70% over 2 min, to 98% over 12 min, followed by a 2 min hold at 98%. The gradient was ramped to 100% of 5.0 mM ammonium acetate, followed by a 5 min post-run equilibration period. The chromatographic method for RSpak JJ-50 2D column used isocratic elution at a constant 0.3 $mL\cdot min^{-1}$ flow rate with 80 % of 50.0 mM ammonium acetate and 80/20 (v/v) methanol/acetonitrile. All peaks were well resolved. Note that PFAS including both linear and branched isomers (i.e., PFHxS and PFOS) were quantified by the sum of both isomer concentrations.

Analytical Quality Control (QC). An instrument blank consisting of 80/20 (v/v) $MeOH/H_2O$ was analyzed after the injection of the highest calibration standard to check the potential carryover between injections. Instrument sensitivity was checked by injecting the calibration standard prior to analysis and at least once every 24 hours.

Text S4. Theoretical calculations.

The bond dissociation energies (BDEs) of selected bonds for representative PFAS examined in this study, as well as the activation energies (labeled in $\Delta G^‡$) of PFCAs, were calculated using ORCA software (Version 4.2.1).[3] All calculations utilized the atom-pairwise dispersion correction with the Becke-Johnson damping scheme (D3BJ),[4,5] and SMD solvation model.[6] For all the simulated molecules, the B3LYP+D3/6-31G** basis set was used for geometry optimizations and frequency calculations, and the B3LYP+D3/6-311++G** basis set was employed for single point energy calculations for optimized molecules.[7-11] The BDE for each bond was calculated following the steps proposed by Bentel et al.[12] $\Delta G^‡$ of the HF elimination step of a previously proposed PFCA degradation mechanism was calculated as well.[13,14] Calculation results are summarized in **Table S8-S10**. Cartesian coordinates (angstroem) of optimized key reaction species and transition states structures involved in this study are shown in **Table S12**, which is placed at the end of this SI file.

Table S1. Chemical names and structures of 19 selected PFAS in this study.

Chemical Name	Abbreviation	Structure	
Perfluorocarboxylic Acids			
Perfluoropropanoic acid	PFPrA	n = 3	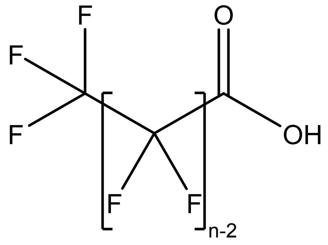
Perfluorobutanoic acid	PFBA	n = 4	
Perfluoropentanoic acid	PFPeA	n = 5	
Perfluorohexanoic acid	PFHxA	n = 6	
Perfluoroheptanoic acid	PFHpA	n = 7	
Perfluorooctanoic acid	PFOA	n = 8	
Perfluorodecanoic acid	PFDA	n = 10	
Perfluorosulfonic Acids			
Perfluoropentane sulfonic acid	PFPeS	n = 5	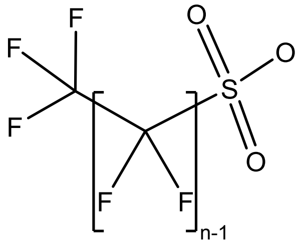
Perfluorohexane sulfonic acid	PFHxS	n = 6	
Perfluorheptane sulfonic acid	PFHpS	n = 7	
Perfluorooctane sulfonic acid	PFOS	n = 8	
Fluorotelomer Sulfonic Acids			
6:2 Fluorotelomer sulfonic acid	6:2 FTS	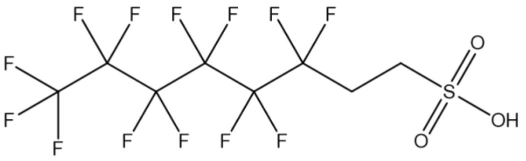	
Fluorotelomer (Unsaturated) Carboxylic Acids			
6:2 Fluorotelomer carboxylic acid	6:2 FTCA	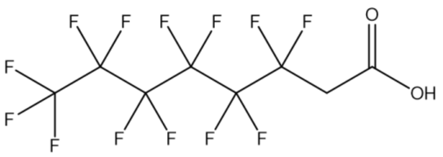	

5:3 Fluorotelomer carboxylic acid	5:3 FTCA	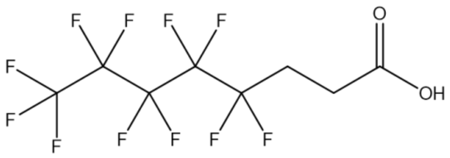
8:2 Fluorotelomer unsaturated carboxylic acid	8:2 FTUCA	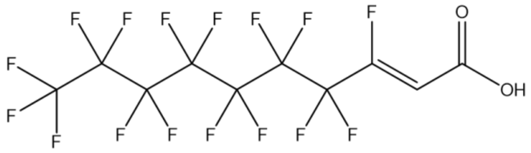
Perfluorosulfonamides		
Perfluorohexane sulfonamide	FHxSA	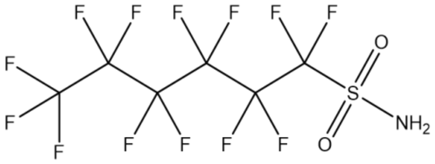
Perfluorooctane sulfonamide	FOSA	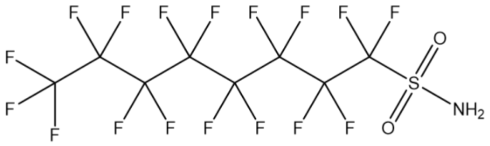
Per- and Polyfluoroethers		
Hexafluoropropylene oxide-dimer acid	HFPO-DA or GenX	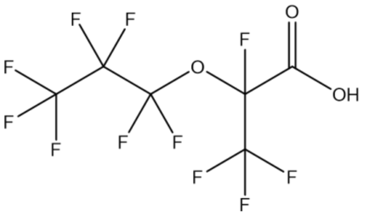
Fluorotelomer Phosphate Diesters		
6:2 Fluorotelomer phosphate diester	6:2 diPAP	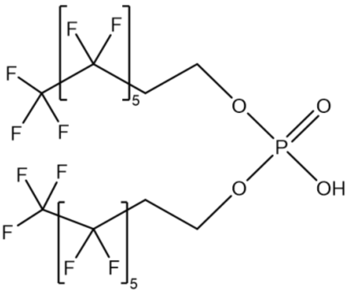

Table S2. PFAS analytes, theoretical m/z, and retention time.

No.	Analyte	Theoretical m/z	Retention Time (min)
1	*TFA	112.9856	6.6
2	*PFPrA	162.9824	5.9
3	PFBA	212.9792	7.5
4	PFPeA	262.976	8.6
5	PFHxA	312.9728	9.3
6	PFHpA	362.9696	10.2
7	PFOA	412.9664	11.1
8	PFNA	462.9632	12.1
9	PFDA	512.9600	13.0
10	PFPeS	348.9398	9.5
11	PFHxS	398.9366	10.4
12	PFHpS	448.9334	11.3
13	PFOS	498.9302	12.2
14	6:2 FTS	426.9679	10.9
15	6:2FTCA	376.9853	10.3
16	5:3FTCA	341.0041	10.2
17	8:2 FTUCA	456.9727	12.8
18	FHxSA	397.9526	11.8
19	FOSA	497.9462	13.9
20	*HFPO-DA (GenX)	284.9779	4.0
21	6:2 diPAP	788.9751	15.6
22	[$^{13}C_2$] 6:2 diPAP	792.9885	15.6

* RSpak JJ-50 2D column (2.0×150 mm, 5 µm) column was used for the detection and quantification of TFA, PFPrA, and HFPO-DA (GenX).

Table S3. Experimental results for PFAS decay and defluorination under 222-nm irradiation at fluence of 4.5×10^{-2} Einstein·L^{-1}.

PFAS	Change in reaction condition	Decay (%)	DeF (%)
Decay and defluorination of selected PFAS			
PFPrA	*	(34 ± 0.6)%	(7 ± 1.6)%
PFBA	*	(51 ± 3)%	(30 ± 0.3)%
PFPeA	*	(57 ± 1)%	(29 ± 0.3)%
PFHxA	*	(65 ± 0.1)%	(30 ± 0.7)%
PFHpA	*	(66 ± 0.6)%	(30 ± 0.1)%
PFOA	*	(67 ± 1)%	(24 ± 3)%
PFDA	*	(69 ± 1)%	(27 ± 0.2)%
PFPeS	*	<10%	negligible
PFHxS	*	<10%	negligible
PFHpS	*	<10%	negligible
PFOS	*	<10%	negligible
6:2 FTS	*	negligible	negligible
6:2 FTCA	*	negligible	negligible
5:3 FTCA	*	negligible	negligible
8:2 FTUCA	*	(81 ± 0.8)%	(31± 0.3)%
FHxSA	*	negligible	negligible
FOSA	*	negligible	negligible
GenX	*	(30 ± 1)%	(17± 0.2)%
6:2 diPAP	*	negligible	negligible
Comparison of direct photolysis under 254-nm and 222-nm irradiation			
PFOA	under 222-nm irradiation, 4 h	(67 ± 1)%	(24 ± 3)%
PFOA	under 254-nm irradiation, 5.6 h	(11 ± 3)%	negligible
Effect of pH			
PFOA	pH = 5.0	(65 ± 3)%	(26 ± 0.05)%

PFOA	pH = 6.0	(67 ± 3)%	(26 ± 0.1)%
PFOA	pH = 7.0	(66 ± 3)%	(25 ± 0.9)%
PFOA	pH = 8.5	(67 ± 1)%	(24 ± 3)%
PFOA	pH = 10.5	(67 ± 3)%	(26 ± 0.9)%
Effect of DO			
PFOA	purged by N_2 gas for 0.5 h	(67 ± 1)%	(24 ± 3)%
PFOA	not purged	(68 ± 0.8)%	(26 ± 2)%
Effect of anions			
PFOA	MQW	(65 ± 2)%	(25 ± 0.14)%
PFOA	5 mM $NaHCO_3$	(67 ± 1)%	(24 ± 3)%
PFOA	5 mM Na_2HPO_4, NaH_2PO_4	(68 ± 0.1)%	(23 ± 0.2)%
PFOA	5 mM NaCl	(74 ± 0.1)%	(32 ± 0.6)%
Effect of real water matrix			
PFOA	in ideal bicornate buffer	(67 ± 1)%	(24 ± 3)%
PFOA	in real wastewater	(25 ± 0.7)%	(7 ± 0.8)%
PFOA	10 ppm HA	(71 ± 1)%	(22 ± 0.9)%
Effect of presence of nitrate			
PFOA	no nitrate	(67 ± 1)%	(24 ± 3)%
PFOA	15 ppm nitrate	(24 ± 4)%	negligible
PFOA	10 ppm nitrate	(21 ± 3)%	negligible
PFOA	5 ppm nitrate	(40 ± 0.4)%	(9 ± 1.4)%
PFOA	0.5 ppm nitrate	(66 ± 1.7)%	(21 ± 0.4)%

* Default reaction conditions: [HCO_3^-] = 5 mM, pH = 8.5, solution was initially purged by N_2 gas for 0.5 h, 4.0 h reaction time under 222-nm irradiation.

Table S4. Fluence-based and time-based rate constants (*k*) of photolysis of PFOA under 222-nm irradiation and various reaction conditions.

	**Fluence-based *k* (L·Einstein^{-1})**	**Time-based *k* (min^{-1})**	**R^2**
Default conditions*	25.02 ± 0.73	(4.71 ± 0.14) × 10^{-3}	0.98
pH			
pH = 5.0	21.01 ± 0.82	(3.96 ± 0.15) × 10^{-3}	0.97
pH = 6.0	22.78 ± 0.55	(4.29 ± 0.10) × 10^{-3}	0.99
pH = 7.0	23.72 ± 0.47	(4.47 ± 0.09) × 10^{-3}	0.99
pH = 10.5	23.11 ± 0.37	(4.35 ± 0.07) × 10^{-3}	1.00
Dissolved oxygen			
With DO	26.02 ± 0.51	(4.90 ± 0.01) × 10^{-3}	1.00
Anoins			
Milli-Q water	22.46 ± 0.69	(4.23 ± 0.13) × 10^{-3}	0.98
HPO_4^{2-}, $H_2PO_4^-$	25.03 ± 0.28	(4.71 ± 0.05) × 10^{-3}	1.00
Cl^-	27.49 ± 0.77	(5.18 ± 0.14) × 10^{-3}	0.99
Real water matrices			
WW Effluent**	4.98 ± 0.37	(0.94 ± 0.07) × 10^{-3}	0.96
10 ppm HA	27.47 ± 0.51	(5.17 ± 0.09) × 10^{-3}	0.99
Presence of nitrate			
0.5 ppm	23.84 ± 0.46	(4.49 ± 0.09) × 10^{-3}	0.99
5 ppm	11.28 ± 0.34	(2.12 ± 0.06) × 10^{-3}	0.99
10 ppm	4.13 ± 0.32	(0.78 ± 0.06) × 10^{-3}	0.91
15 ppm	6.22 ± 0.52	(1.17 ± 0.10) × 10^{-3}	0.90

*Default conditions: [HCO_3^-] = 5 mM, pH = 8.5, solution was initially purged by N_2 gas for 0.5 h, 4 h reaction under 222-nm irradiation.

** Tertiary wastewater effluent

Table S5. Results of quantification of products and mass balance of fluorine (F) for PFAS degradation under 222-nm irradiation at fluence of 4.5×10^{-2} Einstein·L^{-1}.

Reaction condition	Parent compound	Main products	F in remaining parent compound	F in products	Released F^- (deF%)	Unknown
Degradable PFAS						
*	PFPrA	TFA	66%	9%	7%	18%
*	PFBA	PFPrA, TFA	49%	7%	30%	14%
*	PFPeA	PFBA, PFPrA	43%	10%	29%	18%
*	PFHxA	PFPeA, PFBA	35%	24%	30%	11%
*	PFHpA	PFHxA, PFPeA, PFBA	35%	27%	30%	8%
*	PFOA	PFHpA, PFHxA, PFPeA, PFBA	33%	39%	24%	4%
*	PFDA	PFNA, PFOA, PFHpA, PFHxA	31%	35%	27%	7%
*	GenX	PFPrA, TFA	70%	11%	17%	2%
*	8:2 FTUCA	PFOA, PFHpA, PFHxA, PFPeA, PFBA	19%	33%	31%	17%
Effect of pH						
pH = 5.0	PFOA	PFHpA, PFHxA, PFPeA, PFBA	35%	31%	26%	8%
pH = 6.0	PFOA		33%	31%	26%	10%
pH = 7.0	PFOA		34%	35%	25%	6%
pH = 8.5	PFOA		33%	39%	24%	4%
pH = 10.5	PFOA		35%	37%	26%	2%
Effect of DO						
purged by N_2 gas for 0.5 h	PFOA	PFHpA, PFHxA, PFPeA,	33%	39%	24%	4%
not purged	PFOA		32%	18%	26%	24%

		PFBA				
Effect of anions						
Milli-Q water	PFOA	PFHpA,	35%	31%	25%	9%
5 mM $NaHCO_3$	PFOA	PFHxA,	33%	39%	24%	4%
5 mM Na_2HPO_4/ NaH_2PO_4		PFPeA, PFBA	32%	9%	23%	36%
5 mM NaCl			26%	18%	32%	24%
Effect of real water matrix						
in default bicornate buffer	PFOA	PFHpA, PFHxA, PFPeA, PFBA	33%	39%	24%	4%
in real wastewater	PFOA	PFHpA, PFHxA, PFPeA	75%	7%	7%	11%
10 ppm HA	PFOA	PFHpA, PFHxA, PFPeA	29%	11%	22%	38%
Effect of presence of nitrate						
no nitrate	PFOA	PFHpA, PFHxA, PFPeA, PFBA	33%	39%	24%	4%
15 ppm nitrate	PFOA	PFHpA, PFHxA	76%	7%	~0%	17%
10 ppm nitrate	PFOA	PFHpA, PFHxA, PFPeA	80%	6%	~0%	14%
5 ppm nitrate	PFOA	PFHpA, PFHxA, PFPeA	60%	16%	9%	15%
0.5 ppm nitrate	PFOA	PFHpA, PFHxA, PFPeA, PFBA	34%	29%	21%	16%

*Default reaction conditions: [HCO_3^-] = 5 mM, pH = 8.5, solution was initially purged by N_2 gas for 0.5 h, 4.0 h reaction time under 222-nm irradiation.

Table S6. UV absorbance at 222-nm wavelength of PFAS stock aqueous solutions and corresponding molar absorption coefficient (ε).

PFAS	Concentration (mM)	UV absorbance at 222 nm	ε ($M^{-1}·cm^{-1}$)
PFDA	0.6	0.0634	105.67
PFOA	1.0	0.0845	84.47
PFHpA	1.0	0.0905	90.55
PFHxA	1.5	0.1280	85.35
PFPeA	1.4	0.0993	70.96
PFBA	1.3	0.0805	61.96
PFPrA	1.8	0.0478	26.66
GenX	12.0	0.3076	25.63
8:2 FTUCA	0.3	0.1561	520.43
PFPeS	0.5	0.0232	46.50
PFHxS	1.0	0.0140	13.99
PFHpS	1.0	0.0274	27.44
PFOS	1.0	0.0395	39.48
6:2 FTS	1.1	0.0404	35.45
6:2 FTCA	0.1	0.0544	543.85
5:3 FTCA	0.1	0.7987	798.69
6:2 diPAP	0.6	0.7915	1319.11
FOSA	1.0	0.0856	85.62
FHxSA	1.0	0.0181	18.14

Table S7. Statistical analysis between molar absorption coefficients (ε) at the 222-nm wavelength and the degradation rate constants (k in L·Einstein^{-1}) and overall decay% of PFCAs and GenX, showing correlation analysis and liner regressions results.

Variable	Correlation coefficients	t-stat	p-value
k	0.94	6.95	4.42×10^{-4}
$k = 0.2\ \varepsilon + 3.23$ (adjusted $R^2 = 0.87$)			
Decay%	0.98	13.79	9.03×10^{-6}
Decay% = 0.51 ε + 19.42 (adjusted $R^2 = 0.96$)			

Table S8. Calculated αC-C and αC-F BDEs (kcal·mol^{-1}) for PFCAs. Simulation conditions: B3LYP+D3/6-31G** as the geometry optimization method, B3LYP+D3/6-311++G** as the single-point energy calculation method, and SMD as the solvation model.

For PFCAs, use PFOA as an example: $C_6F_{13} - \overbrace{CF_2}^{\alpha C-F} \underbrace{-}_{\alpha C-C} COO^-$

PFCAs	# of C	αC-C	αC-F
PFPrA	3	85.70	111.93
PFBA	4	86.70	110.87
PFPeA	5	85.03	111.99
PFHxA	6	84.54	111.64
PFHpA	7	84.47	111.68
PFOA	8	83.39	110.54
PFDA	10	82.63	110.50

Table S9. Calculated BDEs (kcal·mol^{-1}) for PFOS, GenX and 8:2 FTUCA. Simulation conditions: B3LYP+D3/6-31G** as the geometry optimization method, B3LYP+D3/6-311++G** as the single-point energy calculation method, and SMD as the solvation model.

For PFOS: $C_7F_{15}-CF_2\underbrace{-}_{C-S}SO_3^-$

For GenX: $C_3F_7\overbrace{-}^{O\_1}O\underbrace{-}_{O\_2}CF(CF_3)COO^-$

For 8:2 FTUCA: $C_7F_{15}-CF\underbrace{=}_{C=C}CH\underbrace{-}_{C-C}COO^-$

**PFAS**	**Bond**	**BDE** (kcal·mol^{-1})
PFOS	C-S	54.49
GenX	C_3F_7-O (O_1)	61.15
GenX	O-CF(CF_3)COO^- (O_2)	79.80
8:2 FTUCA	C-C	97.26
8:2 FTUCA	C=C	170.39

Table S10. Calculated activation energies ($\Delta G^{\ddagger}$, kcal·mol^{-1}) for PFCAs. Simulation conditions: B3LYP+D3/6-31G** as the geometry optimization method, B3LYP+D3/6-311++G** as the single-point energy calculation method, and SMD as the solvation model.

Activation energies for the HF elimination step in the previously proposed PFCA (n = 1-6, 8) degradation mechanism[13,14] were calculated (circled in red):

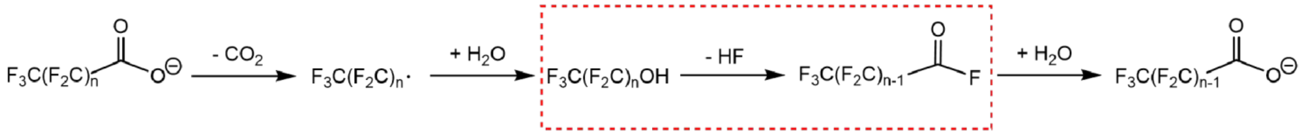

**PFCAs**	**n**	**$\Delta G^{\ddagger}$** (kcal·mol^{-1})
PFPrA	1	40.85
PFBA	2	39.97
PFPeA	3	39.36
PFHxA	4	39.20
PFHpA	5	38.86
PFOA	6	38.51
PFDA	8	37.32

Table S11. Statistical analysis between activation energies ($\Delta G^{\ddagger}$) for the HF elimination step and the degradation rate constants (k in L·Einstein^{-1}) and overall decay% of PFCAs and GenX, showing correlation analysis and liner regressions results.

**Variable**	**Correlation coefficients**	**t-stat**	**p-value**
k	-0.88	-4.21	8.37×10^{-3}
$k = -4.45 \Delta G^{\ddagger} + 192.83$ (adjusted $R^2 = 0.78$)			
Decay%	-0.90	-4.56	6.03×10^{-3}
Decay% $= -10.06 \Delta G^{\ddagger} + 452.20$ (adjusted $R^2 = 0.77$)			

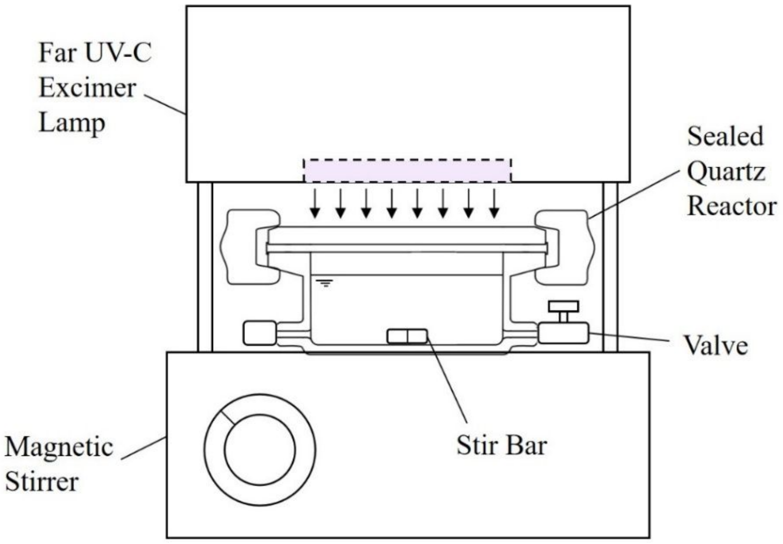

Figure S1. Illustration of experimental set-up using 222-nm KrCl* excimer lamp.

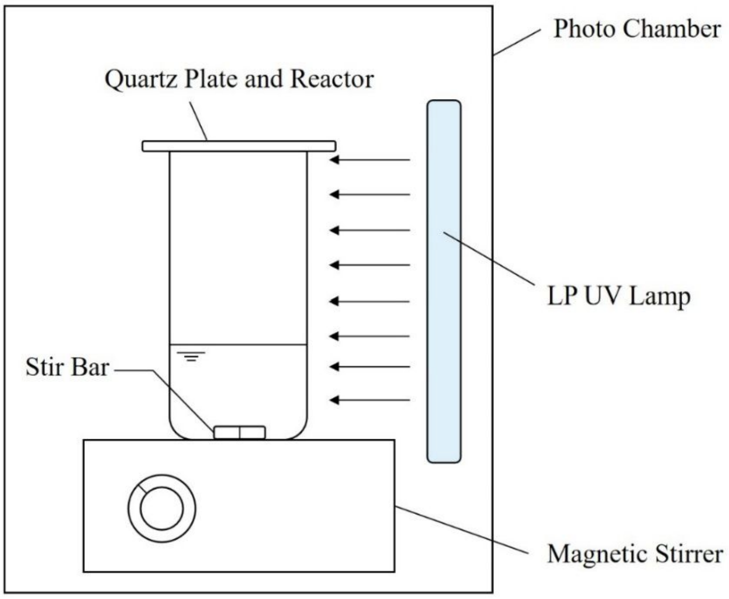

Figure S2. Illustration of experimental set-up using 254-nm LPUV mercury lamp.

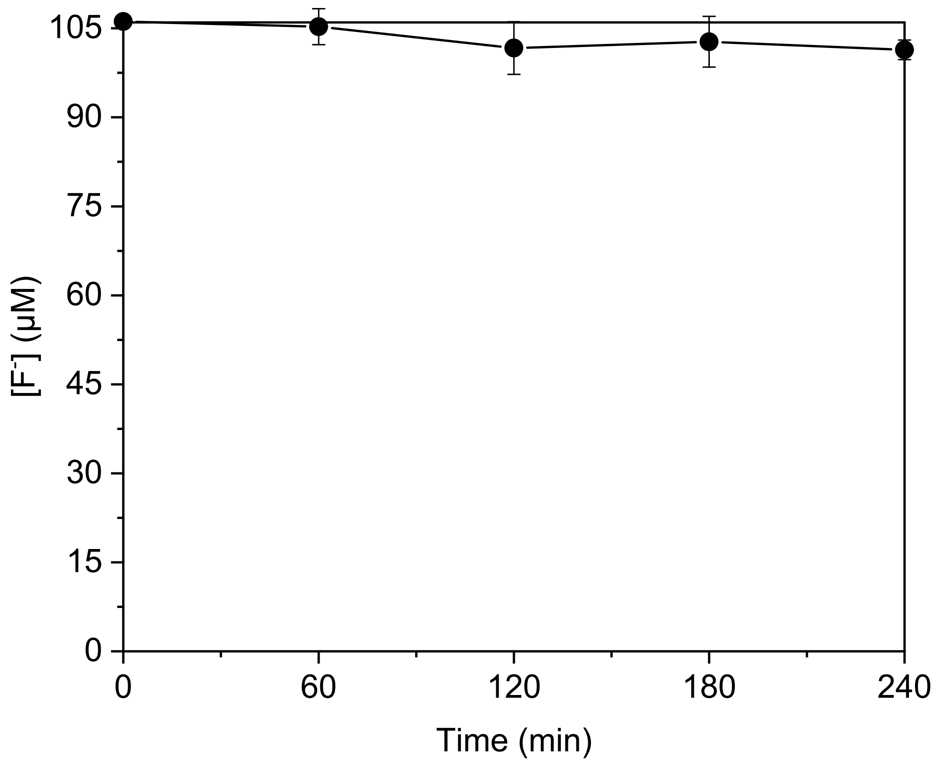

Figure S3. The adsorption of F^- on the quartz reactor: change in concentration for 4 h irradiation at 222 nm. Reaction condition: $[HCO_3^-]$ = 5 mM, initial pH = 8.5, initial $[F^-]$ = 106.1 ± 0.88 μM; final $[F^-]$ = 101.4 ± 1.65 μM.

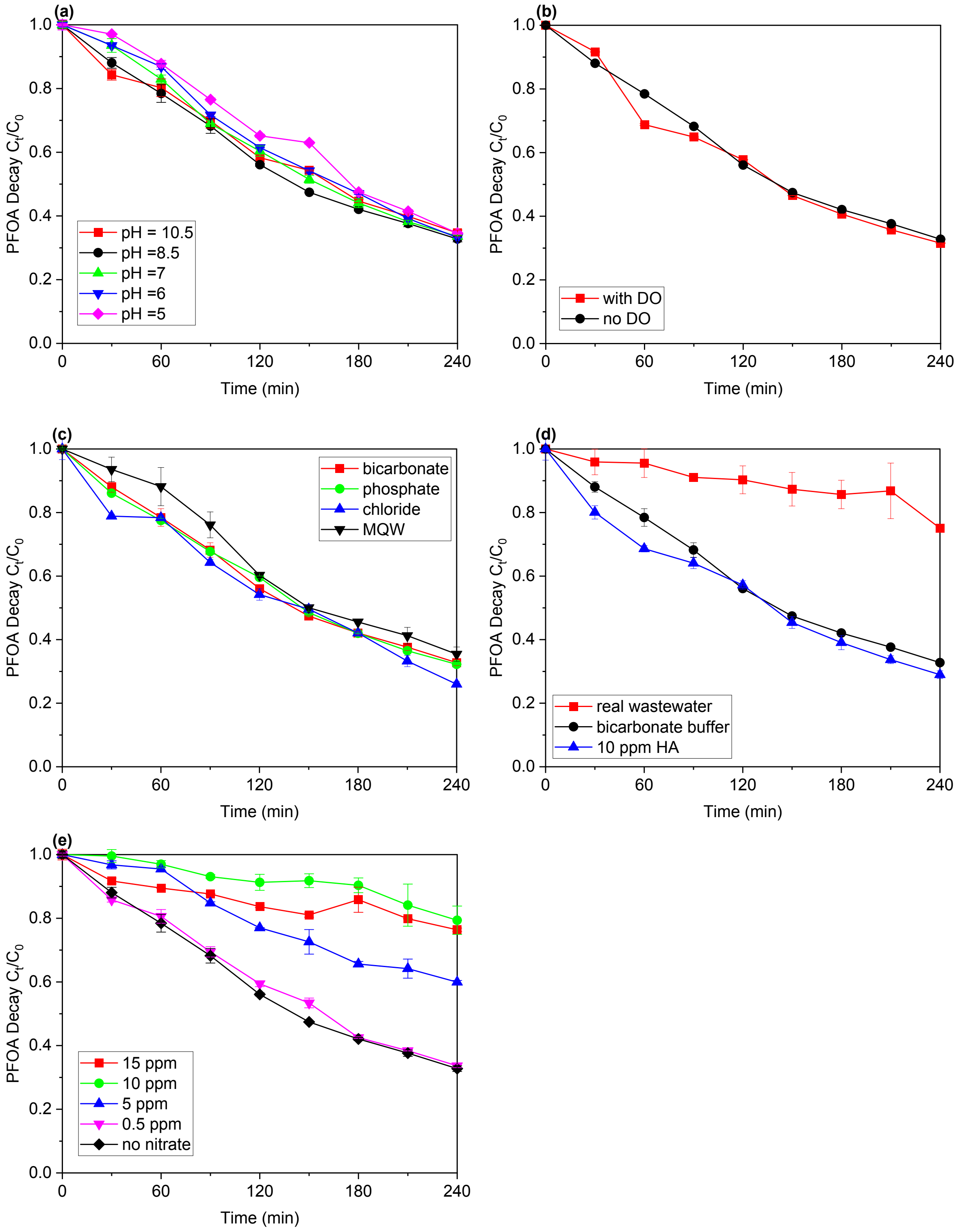

Figure S4. Effect of different reaction conditions on the photodegradation of PFOA under 222-nm irradiation: (a) solution pH; (b) the presence of dissolved oxygen; (c) the presence of different anions; (d) real water matrix; (e) the presence of nitrate. Reaction conditions: $[PFOA]_0$ = 25–35 μM; initial pH = 8.5 for (b) and 7.0 -7.5 for (c) –(e). (a) initial pH = 5, 6, 7, 8.5 and 10.5; (c) [anions] = 5 mM; (e) $[NO_3^-]$ = 0, 0.5, 5, 10, 15 mg·L^{-1}; $[HCO_3^-]$ = 5 mM for (a), (b), (d) and (e). In (c) and (d), experiments were conducted in the absence of HCO_3^- for anion effects and real water matrix effect.

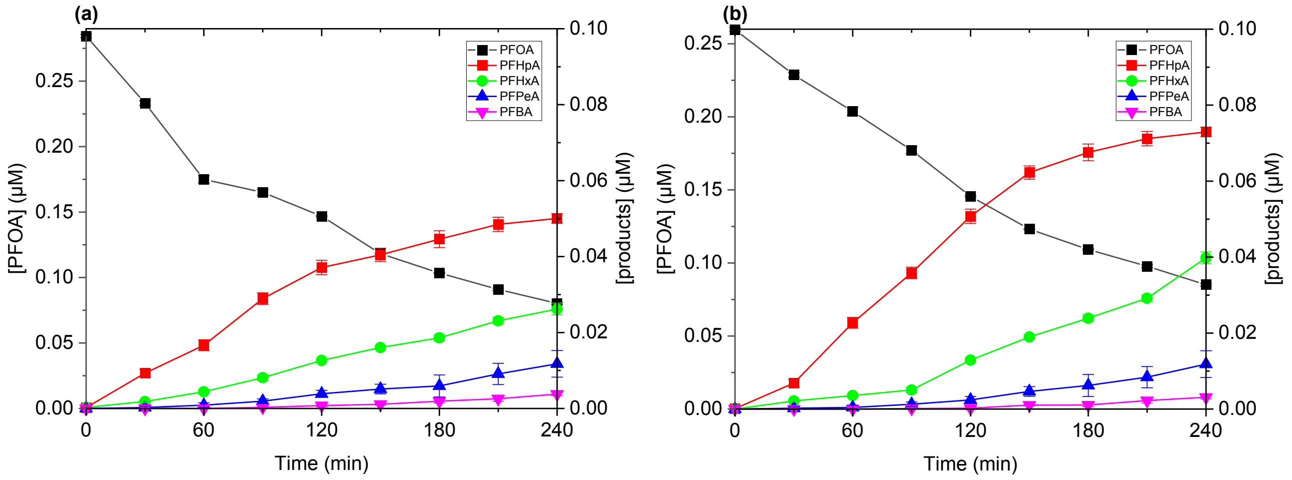

Figure S5. Representative degradation products of PFOA uder 222-nm irradiation with the presence of dissolved oxygen (DO): (a) with DO; (b) without DO. Reaction condition: $[PFOA]_0 = 25.0 \pm 1.2$ μM, $[HCO_3^-]$ = 5 mM, initial pH = 8.5.

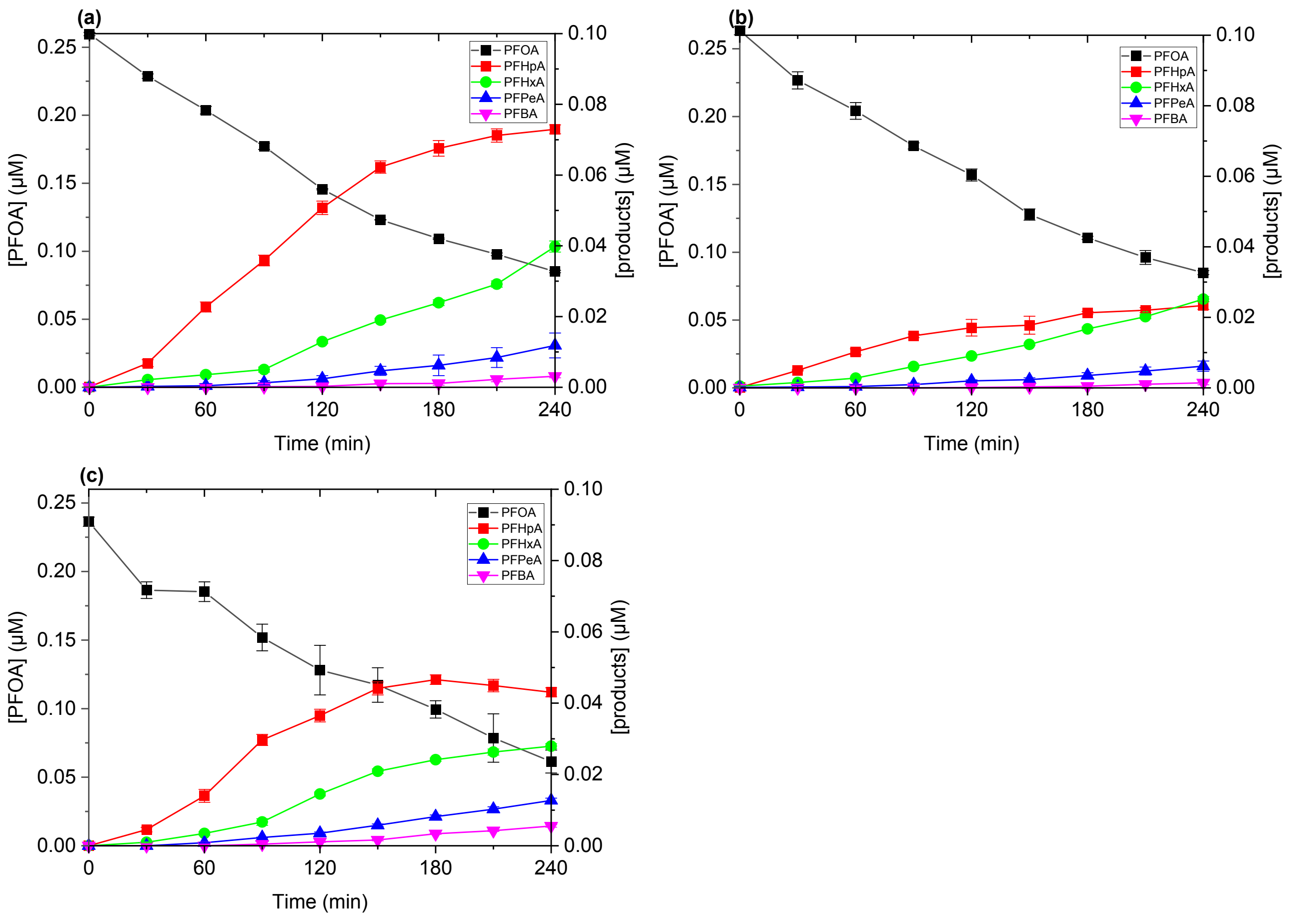

Figure S6. Representative degradation products of degradable PFOA with the presence of different anions: (a) $[HCO_3^-]$ = 5 mM; (b) $[HPO_4^{2-} + H_2PO_4^-]$ = 5 mM; (c) $[Cl^-]$ = 5 mM. Reaction condition: $[PFOA]_0$ = 25.0 ± 1.3 μM, initial pH = 8.5 (a) and 7.5 (b and c).

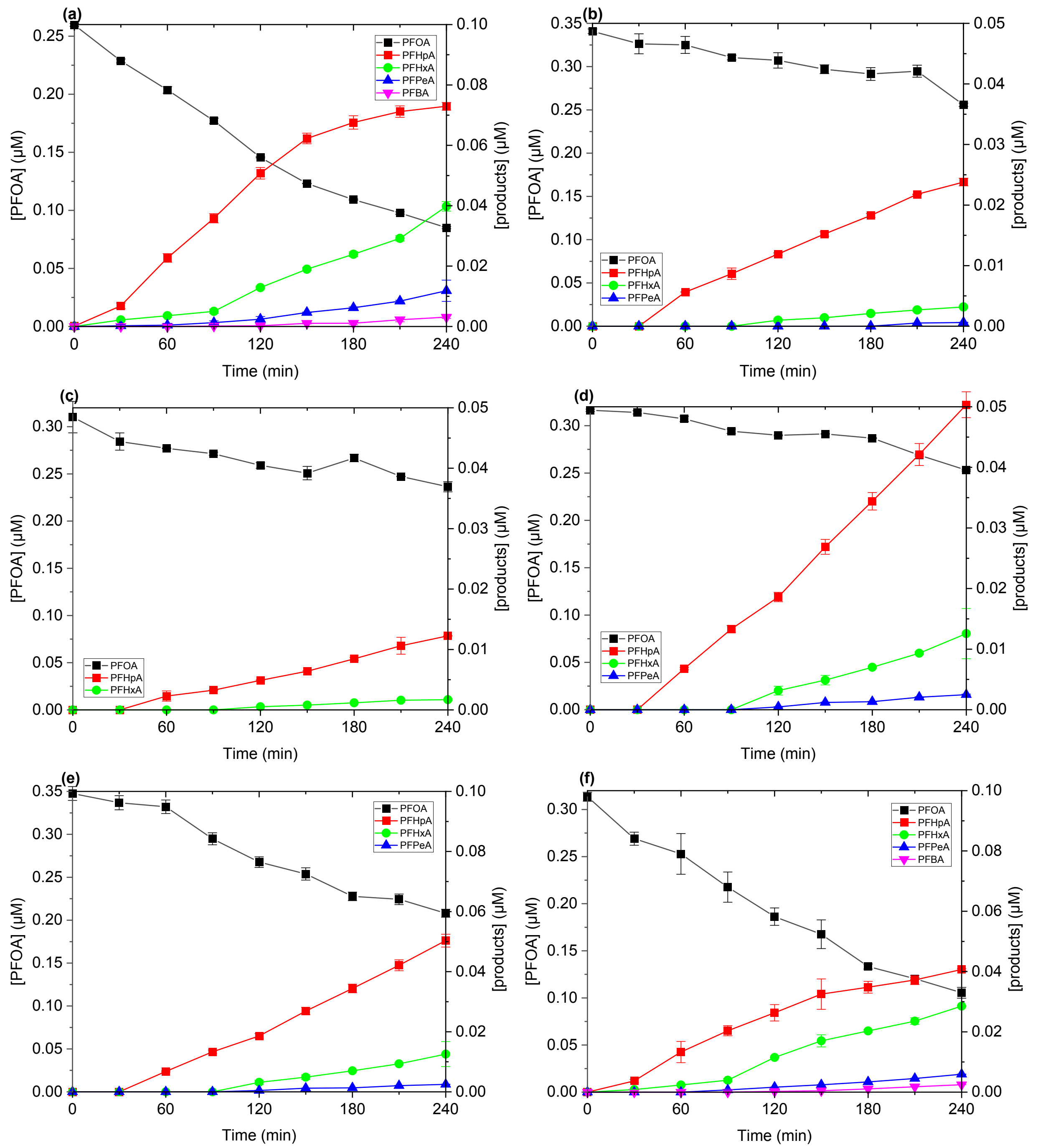

Figure S7. Representative degradation products of degradable PFOA: (a) in ideal buffered solution; (b) real water matrix; (c)-(f) with the presence of nitrate. Reaction condition: $[PFOA]_0$ = 25–35 μM, initial pH = 8.5 (a) and 7.5 (b)-(f). (a) $[HCO_3^-]$ = 5 mM; (c)-(f) $[NO_3^-]$ = 0, 0.5, 5, 10, 15 mg·L^{-1}.

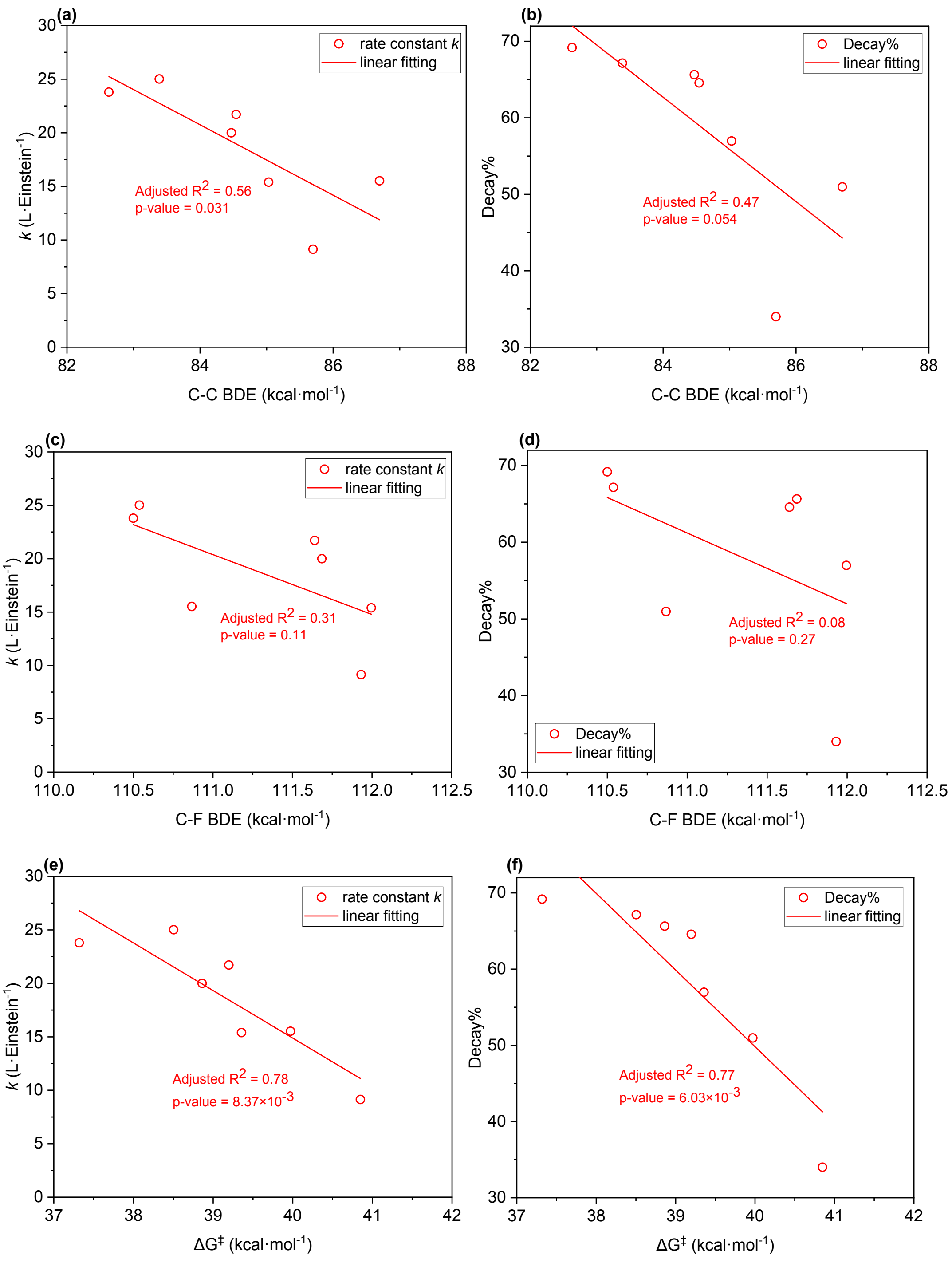

Figure S8. Correlation of the fluence-based rate constants (*k*) and overall decay% of PFCAs (C3-C10) with (a)-(b) C-C BDEs; (c)-(d) C-F BDEs; (e)-(f) activation energies.

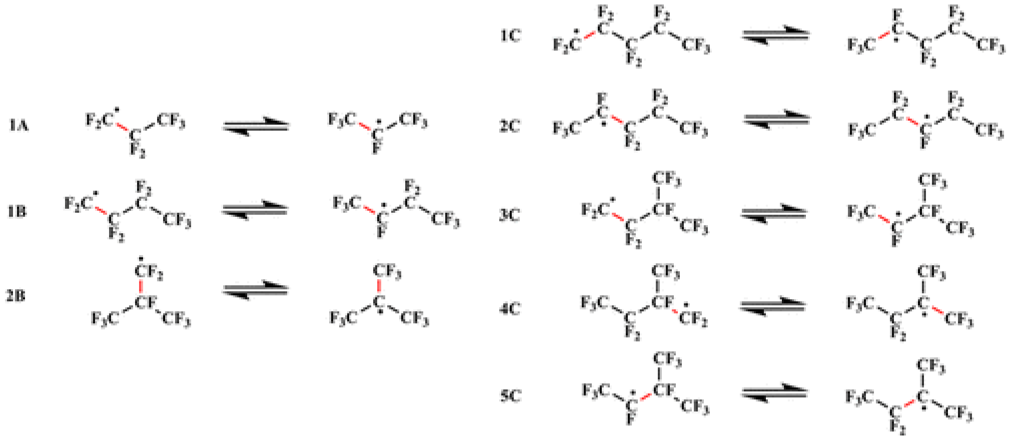

Figure S9. Scheme of 1,2-F atom rearrangements in perfluoropropyl (1A), perfluorobutyl (1B, 2B), and perfluoropentyl (1C–5C) radical systems (adapted from Scheme 1 of Van Hoomissen et al.)[15]

Table S12. Cartesian coordinates of optimized PFAS molecules and reaction species.

**Functional group structures**							
·COO⁻				·SO_3^-			
C	0.039115	0.256425	-0.287292	S	2.685543	-2.924145	-0.351602
O	-0.907444	0.613973	0.450766	O	2.571547	-2.457057	1.082902
O	0.831174	-0.713550	-0.269473	O	3.961223	-3.684138	-0.641257
				O	1.440529	-3.613858	-0.864401
**PFPrA and related structures**							
$C_2F_5COO^-$				C_2F_5·			
C	1.819827	-0.978133	-0.683842	C	0.850989	-0.401622	0.380908
C	3.319361	-1.259075	-0.501825	F	1.481609	0.669919	0.846461
C	4.198438	-0.001795	-0.116293	F	0.932357	-1.434460	1.211145
O	3.638147	0.720327	0.735963	C	-0.550721	-0.121671	-0.117304
O	5.299650	0.052855	-0.691034	F	-0.515546	0.856463	-1.032389
F	3.721251	-1.859872	-1.667937	F	-1.062232	-1.229959	-0.670054
F	3.401324	-2.225011	0.483153	F	-1.370289	0.266683	0.882262
F	1.184112	-2.060259	-1.219033				
F	1.623171	0.046116	-1.539806				
F	1.191698	-0.698851	0.466085				
CF_3CF·(COO^-)				C_2F_5OH			
C	1.806330	-0.954594	-0.708948	C	1.816789	-1.040832	-0.750135
C	3.296053	-1.020634	-0.745131	C	3.304625	-1.122571	-0.338383
C	4.238549	-0.013196	-0.120837	F	3.948709	-1.828715	-1.310767
O	3.617419	0.892562	0.495314	F	3.406599	-1.801156	0.814997
O	5.457032	-0.215303	-0.312291	F	1.330192	-2.237401	-1.086152
F	3.737559	-1.877123	-1.697979	F	1.717274	-0.215332	-1.819714
F	1.254424	-2.097241	-1.221790	O	3.861867	0.102284	-0.154942

F	1.274325	0.065044	-1.446032	H	3.712892	0.630745	-0.952942
F	1.313964	-0.818201	0.539971	F	1.081856	-0.532216	0.244167
C_2F_5OH (TS)							
C	1.705198	-1.044460	-0.716356				
C	3.124780	-0.833986	-0.127167				
F	4.133933	-1.294592	-1.541259				
F	3.383143	-1.718078	0.807520				
F	1.483987	-2.320553	-1.021584				
F	1.528603	-0.287157	-1.793135				
O	3.707695	0.281073	-0.120263				
H	4.288391	-0.161681	-1.071513				
F	0.825072	-0.665761	0.229887				

PFBA and related structures

$C_3F_7COO^-$				$C_3F_7\cdot$			
C	0.966328	-0.349664	0.278182	C	0.922195	-0.320666	0.265627
C	1.900725	-0.901958	-0.816534	C	1.795095	-0.857072	-0.849676
F	1.211810	-1.929505	-1.419598	F	1.545366	-2.117341	-1.183394
F	2.011234	0.093881	-1.763792	F	1.875605	-0.064658	-1.911570
F	1.321929	0.909489	0.630315	F	1.363228	0.915286	0.599966
F	1.021558	-1.148809	1.381761	F	1.027668	-1.150386	1.330660
C	-0.532089	-0.272309	-0.102900	C	-0.579731	-0.201768	-0.087995
F	-0.711063	0.354100	-1.279176	F	-0.733048	0.621309	-1.135388
F	-1.097023	-1.487332	-0.177738	F	-1.063711	-1.409398	-0.412596
F	-1.209358	0.428122	0.836462	F	-1.268616	0.280710	0.951347
C	3.333571	-1.332416	-0.288652				
O	3.870620	-0.421938	0.375081				
O	3.688435	-2.473586	-0.627867				

$CF_3CF_2CF\cdot(COO^-)$				C_3F_7OH			
C	0.992355	-0.293483	0.372362	C	1.869658	-0.993307	-0.804723
C	1.944413	-0.913561	-0.588096	C	3.344019	-1.096244	-0.325651
F	1.363896	-1.261747	-1.765014	F	4.070772	-1.574577	-1.380572
F	1.367754	0.977508	0.741390	F	3.397634	-2.002908	0.662783
F	0.874366	-1.012908	1.539843	F	1.380819	-2.234474	-1.021188
C	-0.453068	-0.146001	-0.152202	O	3.855820	0.063557	0.152904
F	-0.509200	0.633484	-1.247881	H	3.735629	0.761716	-0.507625
F	-1.001796	-1.338133	-0.448630	F	1.142567	-0.390567	0.164701
F	-1.231215	0.430401	0.790229	C	1.674501	-0.184397	-2.107919
C	3.414470	-1.173781	-0.334802	F	2.347281	0.996888	-2.015250
O	3.764893	-0.789740	0.810829	F	2.141216	-0.847083	-3.164850
O	4.037998	-1.714458	-1.272885	F	0.383046	0.089615	-2.293506
C_3F_7OH (TS)							
C	1.787325	-0.959424	-0.795743				
C	3.169516	-0.800246	-0.107252				
F	4.293278	-1.558987	-1.271601				
F	3.234523	-1.577652	0.951192				
F	1.571810	-2.245767	-1.111409				
O	3.870380	0.245332	-0.161858				
H	4.541123	-0.404683	-0.921563				
F	0.869212	-0.578626	0.130690				
C	1.562662	-0.075616	-2.061072				
F	1.611219	1.219090	-1.735976				
F	2.479844	-0.326263	-2.996907				
F	0.352070	-0.348938	-2.559397				
**PFPeA and related structures**							

$C_4F_9COO^-$

C	0.929254	-0.460618	0.316318
C	1.846453	-1.032715	-0.789488
C	3.332074	-1.174292	-0.401542
C	4.092124	0.183826	-0.079585
O	4.456250	0.296612	1.103470
O	4.187324	0.911847	-1.087009
F	3.913314	-1.818852	-1.471003
F	3.345892	-2.064787	0.644355
F	1.334860	-2.270139	-1.098651
F	1.698483	-0.253652	-1.889137
F	1.430844	0.694318	0.805645
F	0.816867	-1.360296	1.326408
C	-0.514929	-0.142602	-0.145536
F	-0.544626	0.891057	-0.996033
F	-1.092933	-1.201432	-0.736853
F	-1.258310	0.184926	0.931080

$C_4F_9\cdot$

C	0.897840	-0.524583	0.303458
C	1.770096	-1.124922	-0.821883
C	3.230398	-1.225429	-0.433337
F	4.003624	-1.690024	-1.406512
F	3.457551	-1.837678	0.724134
F	1.270525	-2.352831	-1.140909
F	1.684015	-0.336739	-1.922462
F	1.520614	0.578258	0.787390
F	0.780722	-1.438182	1.292895
C	-0.527338	-0.107107	-0.132469
F	-0.476634	0.949150	-0.951086
F	-1.139165	-1.120671	-0.759002
F	-1.235005	0.221674	0.955346

$CF_3C_2F_4CF\cdot(COO^-)$

C	0.959434	-0.154579	0.210547
C	1.825179	-0.545078	-1.027370
C	3.236974	-0.944294	-0.765266
C	4.228939	-0.157024	0.052208
O	3.666922	0.817608	0.625295
O	5.410521	-0.554784	0.040042
F	3.660379	-1.879419	-1.646429
F	1.170250	-1.606170	-1.631402
F	1.706787	0.503010	-1.920440

C_4F_9OH

C	0.853137	-0.548302	0.373892
C	1.809240	-1.022433	-0.745596
C	3.305321	-1.117000	-0.327899
F	3.917059	-1.901206	-1.258868
F	3.404281	-1.733660	0.863106
F	1.380930	-2.218544	-1.191532
F	1.747669	-0.121382	-1.777602
F	1.394603	0.522486	1.001520
F	0.698396	-1.556545	1.258683

F	0.968611	1.174939	0.429069	C	-0.554849	-0.130694	-0.121297
F	1.373064	-0.814698	1.319788	F	-0.486503	1.016820	-0.807446
C	-0.541350	-0.523350	0.048857	F	-1.078191	-1.080678	-0.907604
F	-1.042804	-0.059621	-1.109261	F	-1.354140	0.046325	0.936751
F	-0.749379	-1.848026	0.109810	O	3.904564	0.094582	-0.225324
F	-1.246479	0.039474	1.052638	H	3.686972	0.613993	-1.013392
C_4F_9OH (TS)							
C	0.748110	-0.471200	0.400724				
C	1.773271	-0.903651	-0.700463				
C	3.225916	-0.880906	-0.147645				
F	4.128952	-1.378987	-1.620404				
F	3.422632	-1.844339	0.722824				
F	1.465149	-2.142133	-1.123211				
F	1.697267	-0.043102	-1.731440				
F	1.203741	0.657928	0.989771				
F	0.683698	-1.447146	1.330735				
C	-0.701515	-0.195479	-0.111718				
F	-0.732495	0.885709	-0.892937				
F	-1.163811	-1.245297	-0.797642				
F	-1.494544	0.014810	0.943504				
O	3.935267	0.157314	-0.110143				
H	4.436849	-0.299761	-1.094562				

PFHxA and related structures

$C_5F_{11}COO^-$				$C_5F_{11}\cdot$			
C	-2.822601	0.745449	0.906666	C	-2.861514	0.766394	0.922449
C	-1.298389	1.002736	0.802894	C	-1.334020	1.013374	0.840745
C	-0.522827	-0.086642	0.012059	C	-0.566186	-0.041845	0.004037

C	1.016044	-0.060403	0.244471	C	0.974894	-0.035020	0.216468
C	1.842621	-0.828726	-0.809358	C	1.741410	-0.808548	-0.838745
F	1.116999	-1.958634	-1.107494	F	1.388996	-2.084467	-0.943900
F	1.836529	-0.050544	-1.946213	F	1.825027	-0.211209	-2.018685
F	1.373439	1.249121	0.281331	F	1.409084	1.248357	0.196467
F	1.216036	-0.607299	1.474531	F	1.234786	-0.589119	1.423855
F	-1.026476	-1.290807	0.391709	F	-1.023550	-1.277410	0.316655
F	-0.813019	0.110488	-1.298175	F	-0.812097	0.196864	-1.307491
F	-0.835118	1.091264	2.073105	F	-0.849751	1.018950	2.104688
F	-1.146520	2.200824	0.190629	F	-1.140188	2.227476	0.281732
F	-3.423555	1.848759	1.391161	F	-3.447924	1.840333	1.464157
F	-3.090003	-0.269795	1.737696	F	-3.124492	-0.300313	1.684194
C	3.332678	-1.139604	-0.355772	F	-3.365162	0.567886	-0.303857
O	3.903812	-0.117273	0.075073				
O	3.684164	-2.319145	-0.519980				
F	-3.352677	0.474437	-0.295470				
$CF_3C_3F_6CF\cdot(COO^-)$				$C_5F_{11}OH$			
C	-2.811231	0.802195	0.779547	C	0.888512	-0.562789	0.417437
C	-1.279946	1.034758	0.836675	C	1.798227	-1.014583	-0.753155
C	-0.453790	-0.016485	0.043444	C	3.299523	-1.183722	-0.375139
C	1.058540	-0.051938	0.397280	F	3.857500	-1.956459	-1.348546
C	1.889297	-0.816628	-0.572692	F	3.399268	-1.849472	0.789428
F	1.165258	-1.745263	-1.249265	F	1.318181	-2.170986	-1.245896
F	1.449714	1.256004	0.487143	F	1.746801	-0.060341	-1.736069
F	1.112103	-0.568211	1.680193	F	1.514499	0.415564	1.111161
F	-1.017270	-1.232606	0.280820	F	0.682353	-1.628208	1.227180
F	-0.623557	0.289896	-1.269480	C	-0.493741	-0.022917	-0.033316

F	-0.943922	1.037218	2.148130	F	-0.330577	1.214141	-0.551344
F	-1.054993	2.266935	0.321296	F	-1.003553	-0.839413	-0.984382
F	-3.439510	1.883155	1.279923	O	3.950257	-0.002290	-0.243096
F	-3.170436	-0.260004	1.512523	H	3.724996	0.560168	-0.998784
C	3.406462	-0.846662	-0.616374	C	-1.531225	0.065419	1.120812
O	3.916415	-0.154028	0.300974	F	-2.529992	0.875452	0.751430
O	3.902781	-1.547223	-1.522370	F	-2.028579	-1.147580	1.381983
F	-3.231776	0.621727	-0.481411	F	-0.968087	0.556638	2.233103
$C_5F_{11}OH$ (TS)							
C	0.824443	-0.489492	0.474000				
C	1.778467	-0.915111	-0.697159				
C	3.254282	-0.964545	-0.210150				
F	4.065810	-1.474734	-1.732194				
F	3.443520	-1.950858	0.636783				
F	1.406920	-2.123561	-1.150602				
F	1.689344	-0.010501	-1.688408				
F	1.416653	0.519032	1.154024				
F	0.685556	-1.542928	1.308148				
C	-0.588770	-0.011448	0.002334				
F	-0.476261	1.229905	-0.509102				
F	-1.042736	-0.844403	-0.959115				
O	4.011612	0.039311	-0.188568				
H	4.444489	-0.418966	-1.203963				
C	-1.663783	0.035507	1.137483				
F	-2.755562	0.647267	0.670145				
F	-1.998079	-1.195663	1.528929				
F	-1.201536	0.719809	2.190221				

PFHpA and related structures							
$C_6F_{13}COO^-$				$C_6F_{13}\cdot$			
C	-3.640391	0.549268	-0.407556	C	-3.676378	0.597973	-0.399271
C	-2.846964	0.764334	0.901361	C	-2.888132	0.756922	0.921473
C	-1.311164	0.989092	0.783616	C	-1.354889	0.991568	0.799797
C	-0.522571	-0.103517	0.014012	C	-0.560274	-0.068950	-0.003291
C	1.016898	-0.061061	0.245533	C	0.979928	-0.024476	0.216505
C	1.846619	-0.830283	-0.805725	C	1.765885	-0.808974	-0.816447
F	1.127392	-1.965940	-1.097129	F	1.440690	-2.094111	-0.890578
F	1.833593	-0.057605	-1.946272	F	1.842379	-0.240067	-2.010778
F	1.365323	1.250280	0.273864	F	1.389711	1.265576	0.165737
F	1.221379	-0.599185	1.478304	F	1.245247	-0.543861	1.437619
F	-1.013978	-1.307641	0.402521	F	-0.990866	-1.306680	0.329974
F	-0.811832	0.081663	-1.302142	F	-0.799114	0.141345	-1.323944
F	-0.861766	1.070399	2.060047	F	-0.874996	1.018802	2.066735
F	-1.137118	2.186661	0.177190	F	-1.159410	2.198402	0.226025
F	-3.356603	1.884183	1.481994	F	-3.386583	1.839960	1.562112
F	-3.097002	-0.287298	1.712973	F	-3.105913	-0.341193	1.676510
F	-4.955972	0.627134	-0.130730	F	-4.986386	0.637740	-0.132405
F	-3.342940	1.493951	-1.308394	F	-3.373854	1.587100	-1.249096
F	-3.396042	-0.655567	-0.930531	F	-3.390186	-0.578208	-0.973742
C	3.339548	-1.129257	-0.353055				
O	3.903383	-0.101723	0.074722				
O	3.698892	-2.306515	-0.515571				
$CF_3C_4F_8CF\cdot(COO^-)$				$C_6F_{13}OH$			
C	-3.559705	0.621320	-0.515660	C	-2.847883	0.742867	0.911223
C	-2.849621	0.775028	0.848625	C	-1.317128	0.972186	0.833408

C	-1.309249	1.002678	0.844079	C	-0.557325	-0.114882	0.026294
C	-0.474045	-0.058932	0.078295	C	0.976747	-0.113652	0.285151
C	1.047913	-0.044124	0.394287	C	1.785615	-0.853286	-0.813439
C	1.870444	-0.824066	-0.570832	C	3.259998	-1.161177	-0.438639
F	1.149184	-1.785776	-1.202676	F	3.803699	-1.777968	-1.525962
F	1.410940	1.273911	0.428534	F	3.307549	-2.019976	0.590162
F	1.144785	-0.513813	1.692004	F	1.151947	-2.001943	-1.131797
F	-0.998054	-1.278351	0.369827	F	1.827661	-0.042924	-1.910370
F	-0.681465	0.199880	-1.243565	F	1.411299	1.172411	0.330062
F	-0.953428	1.038331	2.150310	F	1.200717	-0.697990	1.480702
F	-1.098292	2.223675	0.297780	F	-1.041699	-1.331116	0.366757
F	-3.397427	1.868376	1.445317	F	-0.788423	0.102728	-1.289063
F	-3.151821	-0.312070	1.594098	F	-0.840703	1.005598	2.099784
F	-4.890427	0.682528	-0.315342	F	-1.107386	2.169445	0.244003
F	-3.214154	1.609299	-1.350463	F	-3.425929	1.840415	1.413192
F	-3.279343	-0.556033	-1.081281	F	-3.127181	-0.294782	1.705966
C	3.386605	-0.825672	-0.648598	O	3.957866	-0.047907	-0.092605
O	3.902019	-0.094907	0.235378	H	3.836613	0.622066	-0.781689
O	3.876431	-1.543967	-1.543958	F	-3.344774	0.509715	-0.311513
$C_6F_{13}OH$ (TS)							
C	-2.973535	0.697217	0.918036				
C	-1.439174	0.991727	0.843166				
C	-0.614669	-0.082840	0.055708				
C	0.927878	-0.003863	0.326544				
C	1.797275	-0.741203	-0.754397				
C	3.248012	-0.948488	-0.236057				
F	4.075050	-1.478676	-1.736267				

F	3.338167	-1.981117	0.567920	
F	1.243985	-1.936835	-1.036528	
F	1.835273	0.012899	-1.863082	
F	1.314654	1.287463	0.361130	
F	1.182829	-0.563251	1.531920	
F	-1.044774	-1.312539	0.409524	
F	-0.838848	0.094023	-1.263287	
F	-0.976231	1.070807	2.110445	
F	-1.278071	2.186725	0.241669	
F	-3.592494	1.771051	1.417580	
F	-3.220201	-0.345777	1.713076	
O	4.078571	-0.005451	-0.152012	
H	4.520222	-0.476839	-1.162317	
F	-3.462637	0.444794	-0.301143	

PFOA and related structures

$C_7F_{15}COO^-$				$C_7F_{15}\cdot$			
C	-3.636404	0.472778	-0.391885	C	-3.687192	0.611383	-0.383987
C	-2.844426	0.721235	0.912192	C	-2.872517	0.796010	0.917390
C	-1.319517	1.013102	0.784594	C	-1.334145	0.973256	0.764769
C	-0.494926	-0.048685	0.006549	C	-0.590052	-0.144392	-0.012056
C	1.046914	0.061086	0.235394	C	0.948135	-0.131436	0.211046
C	1.859368	-0.716057	-0.848620	C	1.748803	-0.907168	-0.864374
C	3.239545	-1.306043	-0.482668	C	3.195777	-1.113251	-0.450109
C	4.332674	-0.252559	-0.077853	F	4.029065	-1.274913	-1.469630
O	3.855891	0.621466	0.682754	F	3.371539	-2.031830	0.491027
O	5.467891	-0.458013	-0.533438	F	1.135992	-2.108682	-1.078999
F	3.589272	-2.057247	-1.575060	F	1.737629	-0.198358	-2.013783

F	3.016657	-2.199753	0.543374	F	1.394984	1.147069	0.206470
F	1.054399	-1.763619	-1.243775	F	1.204734	-0.683093	1.419652
F	1.994261	0.112188	-1.916449	F	-1.061489	-1.348278	0.380852
F	1.320797	1.382604	0.188013	F	-0.846263	0.027498	-1.332076
F	1.273260	-0.423386	1.473531	F	-0.832922	1.027759	2.022936
F	-0.936372	-1.270889	0.394702	F	-1.107054	2.151237	0.145070
F	-0.792321	0.135032	-1.308127	F	-3.322352	1.921441	1.519968
F	-0.864093	1.113377	2.055691	F	-3.115350	-0.261362	1.720982
F	-1.200951	2.214447	0.174723	F	-4.990196	0.711199	-0.099422
F	-3.395313	1.818016	1.496426	F	-3.362262	1.552809	-1.278531
F	-3.041686	-0.340358	1.724879	F	-3.453474	-0.597274	-0.912330
F	-4.951306	0.488874	-0.106799				
F	-3.387075	1.431621	-1.292386				
F	-3.338694	-0.718699	-0.919436				
$CF_3C_5F_{10}CF\cdot(COO^-)$				$C_7F_{15}OH$			
C	-3.647777	0.421182	-0.322204	C	-3.653484	0.604554	-0.411280
C	-2.809481	0.666982	0.952943	C	-2.870580	0.761662	0.913037
C	-1.302460	1.016626	0.770012	C	-1.331103	0.958059	0.800702
C	-0.474978	0.003587	-0.068122	C	-0.557817	-0.133045	0.013883
C	1.069090	0.160006	0.100793	C	0.975982	-0.115320	0.279508
C	1.872225	-0.588351	-1.012678	C	1.794025	-0.857626	-0.811251
C	3.209227	-1.127267	-0.632167	C	3.268205	-1.155158	-0.427353
C	4.302191	-0.351337	0.056547	F	3.820579	-1.774463	-1.508857
O	3.885665	0.783627	0.421092	F	3.315096	-2.008427	0.606040
O	5.411910	-0.909717	0.156741	F	1.168339	-2.011564	-1.126142
F	3.486709	-2.274592	-1.291415	F	1.836761	-0.053430	-1.912661
F	1.071305	-1.643670	-1.414632	F	1.401724	1.173326	0.315884

F	1.911190	0.271496	-2.092906	F	1.199856	-0.689424	1.479685
F	1.297657	1.489604	0.080635	F	-1.029960	-1.350424	0.359627
F	1.359268	-0.348806	1.320663	F	-0.780475	0.075558	-1.307449
F	-0.860725	-1.242109	0.307745	F	-0.860007	0.988879	2.070829
F	-0.835490	0.208087	-1.363366	F	-1.103309	2.153146	0.213827
F	-0.798881	1.102832	2.024042	F	-3.345722	1.866103	1.534676
F	-1.252208	2.235993	0.187177	F	-3.120906	-0.319336	1.682033
F	-3.373887	1.729007	1.586971	F	-4.963672	0.679016	-0.153489
F	-2.935086	-0.420015	1.746258	F	-3.321676	1.576275	-1.270523
F	-4.948687	0.379297	0.020125	F	-3.391603	-0.584552	-0.969725
F	-3.475208	1.411757	-1.206377	O	3.958086	-0.036340	-0.083556
F	-3.330379	-0.743949	-0.894925	H	3.838354	0.628816	-0.777573
$C_7F_{15}OH$ (TS)							
C	-3.768827	0.557166	-0.421950				
C	-2.978995	0.759651	0.908423				
C	-1.426924	0.995574	0.811336				
C	-0.596438	-0.083890	0.041154				
C	0.944769	-0.007210	0.326848				
C	1.819156	-0.743783	-0.751214				
C	3.264511	-0.960911	-0.222150				
F	4.102736	-1.484249	-1.718441				
F	3.343424	-1.999488	0.575327				
F	1.262213	-1.935265	-1.043757				
F	1.869935	0.015530	-1.855811				
F	1.337510	1.281344	0.370489				
F	1.188103	-0.573987	1.530881				
F	-1.028786	-1.311338	0.393577				

F	-0.804056	0.093377	-1.282644				
F	-0.978492	1.049393	2.086739				
F	-1.221973	2.193187	0.228660				
F	-3.482029	1.859470	1.511255				
F	-3.212121	-0.307486	1.698186				
F	-5.076702	0.569998	-0.152302				
F	-3.495831	1.537503	-1.288138				
F	-3.460010	-0.617893	-0.979379				
O	4.097975	-0.021744	-0.124104				
H	4.547549	-0.488670	-1.133114				

PFDA and related structures

$C_9F_{19}COO^-$				$C_9F_{19}\cdot$			
C	-3.635440	0.269945	-0.237985	C	-3.682526	0.281289	-0.234756
C	-2.801755	0.619604	1.022629	C	-2.831161	0.631294	1.014048
C	-1.296172	0.973602	0.802703	C	-1.317347	0.914466	0.760201
C	-0.453671	-0.098443	0.056252	C	-0.522798	-0.204580	0.036039
C	1.088883	0.096455	0.213968	C	1.018480	-0.051851	0.170619
C	1.898236	-0.716128	-0.846628	C	1.827441	-0.854276	-0.878769
C	3.311794	-1.227942	-0.489274	C	3.305380	-0.904900	-0.530307
C	4.373776	-0.109945	-0.188813	F	4.099646	-1.081343	-1.578036
O	3.878499	0.809346	0.503754	F	3.605535	-1.723893	0.469350
O	5.509520	-0.317314	-0.642035	F	1.309476	-2.115424	-0.954687
F	3.658915	-2.033828	-1.542728	F	1.699346	-0.254510	-2.081903
F	3.156181	-2.061412	0.597849	F	1.357453	1.252305	0.033742
F	1.125422	-1.817568	-1.149287	F	1.379712	-0.472880	1.404802
F	1.966070	0.046214	-1.968454	F	-0.871923	-1.402337	0.554913
F	1.296024	1.422802	0.067261	F	-0.856054	-0.172369	-1.276956

F	1.384144	-0.290797	1.471822	F	-0.766250	1.102342	1.984956
F	-0.819931	-1.311716	0.538363	F	-1.211385	2.055725	0.049216
F	-0.810140	-0.015274	-1.253187	F	-3.332526	1.764647	1.564195
F	-0.797124	1.168177	2.046444	F	-2.934261	-0.374541	1.906690
F	-1.259425	2.141457	0.124306	F	-3.348154	1.107744	-1.250517
F	-3.369167	1.721017	1.588095	F	-3.402178	-0.996328	-0.591020
F	-2.898565	-0.406132	1.894740	C	-5.212133	0.409213	0.004313
F	-3.346358	1.134216	-1.235652	F	-5.540818	1.718319	-0.013869
F	-3.327462	-0.988588	-0.631655	F	-5.525920	-0.116006	1.211132
C	-5.170284	0.337873	0.000730	C	-6.073969	-0.313658	-1.062539
F	-5.557981	1.632682	0.005294	F	-5.665217	0.020764	-2.294326
F	-5.481615	-0.223724	1.192918	F	-5.987925	-1.639301	-0.914145
C	-6.003707	-0.393575	-1.082100	F	-7.350643	0.057036	-0.912670
F	-5.613726	-0.025159	-2.309708				
F	-5.882171	-1.719479	-0.960768				
F	-7.297030	-0.068524	-0.927674				
$CF_3C_7F_{14}CF\cdot(COO^-)$				$C_9F_{19}OH$			
C	-3.634412	0.266521	-0.228641	C	-3.656462	0.282814	-0.229603
C	-2.770968	0.599002	1.016591	C	-2.836375	0.593407	1.050106
C	-1.280597	0.992397	0.762871	C	-1.315197	0.878255	0.844233
C	-0.438656	-0.038900	-0.040022	C	-0.506101	-0.222987	0.108278
C	1.102440	0.183682	0.076792	C	1.034311	-0.079775	0.284806
C	1.906228	-0.601602	-1.011069	C	1.848042	-0.866197	-0.777729
C	3.267378	-1.075049	-0.628988	C	3.359549	-1.018693	-0.457456
C	4.348106	-0.220966	-0.018275	F	3.895843	-1.712969	-1.500847
O	3.901141	0.922202	0.278919	F	3.528134	-1.748750	0.654475
O	5.477604	-0.735476	0.093998	F	1.296736	-2.087141	-0.944113

F	3.567410	-2.253477	-1.219437	F	1.771695	-0.174007	-1.951068
F	1.132770	-1.702759	-1.336491	F	1.369653	1.231625	0.179658
F	1.895725	0.196945	-2.137981	F	1.357935	-0.521640	1.517648
F	1.279700	1.517051	-0.029295	F	-0.871697	-1.433610	0.583263
F	1.442216	-0.238454	1.316881	F	-0.807999	-0.150542	-1.211160
F	-0.765735	-1.273239	0.418613	F	-0.796602	1.029949	2.087467
F	-0.839557	0.073243	-1.333872	F	-1.187364	2.039158	0.168753
F	-0.749086	1.161830	1.997009	F	-3.348280	1.712625	1.619132
F	-1.289683	2.180254	0.119143	F	-2.964978	-0.436782	1.910815
F	-3.345727	1.670864	1.629808	F	-3.294650	1.138691	-1.211258
F	-2.819879	-0.452892	1.861265	F	-3.369935	-0.984120	-0.617029
F	-3.396801	1.167439	-1.207622	C	-5.191183	0.407867	-0.025452
F	-3.310223	-0.971931	-0.669401	F	-5.516456	1.717875	-0.009322
C	-5.162500	0.290296	0.056812	F	-5.536297	-0.155382	1.155358
F	-5.579442	1.574503	0.119364	C	-6.027875	-0.277470	-1.136217
F	-5.425712	-0.320599	1.236493	F	-5.590153	0.097305	-2.346323
C	-6.011463	-0.421337	-1.027161	F	-5.946470	-1.607417	-1.030564
F	-5.668338	0.001012	-2.251376	F	-7.307381	0.090192	-1.003886
F	-5.855553	-1.747449	-0.959384	O	3.981411	0.175745	-0.277320
F	-7.306829	-0.133857	-0.822216	H	3.772545	0.751453	-1.027818
$C_9F_{19}OH$ (TS)							
C	-3.736406	0.285707	-0.228491				
C	-2.914756	0.599349	1.065369				
C	-1.379886	0.911673	0.876971				
C	-0.525158	-0.167607	0.132851				
C	1.021112	0.017815	0.329048				
C	1.884777	-0.739490	-0.742914				

C	3.367226	-0.824327	-0.282492				
F	4.161945	-1.394682	-1.786176				
F	3.558584	-1.797445	0.576089				
F	1.395137	-1.982436	-0.918501				
F	1.826152	-0.062363	-1.899215				
F	1.336772	1.326682	0.265862				
F	1.358808	-0.449339	1.552921				
F	-0.861237	-1.390841	0.589863				
F	-0.809311	-0.097457	-1.186142				
F	-0.882464	1.055331	2.127794				
F	-1.262027	2.085800	0.229218				
F	-3.440337	1.703706	1.644420				
F	-3.034930	-0.433680	1.920607				
F	-3.385558	1.154910	-1.198854				
F	-3.426057	-0.966900	-0.632932				
C	-5.288121	0.384126	-0.026789				
F	-5.639471	1.684937	-0.011930				
F	-5.631016	-0.176445	1.153076				
C	-6.122187	-0.323448	-1.145564				
F	-5.706650	0.063426	-2.357055				
F	-6.017380	-1.650468	-1.050554				
F	-7.406901	0.014972	-1.002014				
O	4.137903	0.171651	-0.294083				
H	4.565835	-0.333677	-1.293557				
**PFOS and related structures**							
$C_8F_{17}SO_3^-$				$C_8F_{17}\cdot$			
C	-3.595392	1.474651	1.400051	C	-3.587987	1.401779	1.450185

C	-2.289658	0.740680	1.797239	C	-2.245195	0.717269	1.808582
C	-0.933084	1.397893	1.412251	C	-0.931430	1.436060	1.393454
C	-0.632487	1.744561	-0.072098	C	-0.680813	1.798169	-0.095976
C	-0.621683	0.638941	-1.160977	C	-0.655980	0.694632	-1.188161
C	0.354412	-0.574997	-1.050316	C	0.376000	-0.464248	-1.097171
C	1.862514	-0.234436	-0.951961	C	1.878875	-0.101362	-1.028957
C	2.871093	-1.363611	-1.278588	C	2.750561	-1.323643	-1.272982
S	2.737140	-2.968305	-0.323228	F	3.941050	-1.259407	-0.691199
O	2.693390	-2.494322	1.077887	F	2.834594	-1.692183	-2.544338
O	3.970099	-3.672510	-0.730493	F	2.142943	0.866000	-1.952129
O	1.472012	-3.532411	-0.845659	F	2.167370	0.375970	0.201329
F	4.093648	-0.800890	-1.055626	F	0.186092	-1.201122	-2.221236
F	2.768279	-1.603969	-2.615003	F	0.079844	-1.233653	-0.024894
F	2.096328	0.784733	-1.840621	F	-0.445020	1.338634	-2.358550
F	2.098595	0.245385	0.290034	F	-1.878317	0.113800	-1.231774
F	0.121879	-1.289522	-2.174150	F	0.512390	2.437131	-0.138492
F	-0.036201	-1.277633	0.031620	F	-1.649909	2.672774	-0.462411
F	-0.408496	1.282692	-2.332822	F	0.083925	0.650220	1.819793
F	-1.877169	0.114783	-1.199308	F	-0.882750	2.608877	2.067337
F	0.558629	2.380601	-0.085367	F	-2.251387	-0.529576	1.288039
F	-1.589678	2.640647	-0.453069	F	-2.196312	0.626915	3.158710
F	0.031531	0.595489	1.894131	F	-3.561387	2.696451	1.788001
F	-0.879247	2.586016	2.075464	F	-4.568366	0.794344	2.129309
F	-2.350124	-0.513141	1.310461	F	-3.843806	1.293141	0.141950
F	-2.286919	0.683203	3.154801				
F	-3.532185	2.775161	1.719730				
F	-4.615407	0.920499	2.076174				

F	-3.847992	1.357590	0.093502				
**GenX and related structures**							
$C_3F_7OCF(CF_3)COO^-$				$\cdot OCF(CF_3)COO^-$			
C	-3.189335	0.300013	-0.485150	O	-0.533031	0.100865	0.798109
C	-2.750524	0.724824	0.933663	C	0.685387	-0.021646	0.987585
C	-1.248832	1.058358	1.104731	F	1.145628	-0.271257	2.302601
F	-1.050227	1.348065	2.418427	C	1.323521	-1.720722	0.020923
F	-1.009745	2.217004	0.419330	O	1.369306	-1.468841	-1.164279
F	-3.472177	1.830020	1.263860	O	1.464726	-2.573674	0.867324
F	-3.085951	-0.275280	1.782241	C	1.647923	1.043579	0.446725
F	-4.539814	0.251447	-0.519746	F	1.588402	2.188223	1.194145
F	-2.783986	1.196083	-1.396699	F	2.944965	0.652831	0.469635
F	-2.720417	-0.902751	-0.805708	F	1.348609	1.395634	-0.815142
O	-0.540230	0.010086	0.649345				
C	0.862939	-0.187325	0.905490				
F	1.048455	-0.252494	2.261303				
C	1.249740	-1.547944	0.148160				
O	1.167164	-1.423338	-1.087489				
O	1.554624	-2.474266	0.912495				
C	1.698361	1.011922	0.427107				
F	1.574901	2.078869	1.265789				
F	3.005328	0.679766	0.424038				
F	1.364153	1.429716	-0.798611				
$C_3F_7\cdot$				$C_3F_7O\cdot$			
C	-3.194805	0.353482	-0.469406	C	-3.210443	0.312207	-0.493308
C	-2.826481	0.784735	0.965374	C	-2.790320	0.729488	0.932642
C	-1.348207	1.036788	1.153038	C	-1.230236	1.045797	1.099297

F	-1.011716	1.339825	2.399989	F	-1.039490	1.343043	2.410048
F	-0.807904	1.870168	0.269397	F	-0.966122	2.176143	0.395005
F	-3.554026	1.900904	1.261726	F	-3.435670	1.860829	1.263716
F	-3.198515	-0.201398	1.815437	F	-3.123337	-0.247619	1.789958
F	-4.481096	-0.007358	-0.515387	F	-4.538314	0.148666	-0.524372
F	-2.993748	1.360789	-1.327965	F	-2.863115	1.251221	-1.378009
F	-2.434510	-0.690152	-0.837255	F	-2.619882	-0.843838	-0.818072
				O	-0.574311	-0.018068	0.687389
$\cdot CF(CF_3)COO^-$							
C	1.250551	-0.282807	0.953196				
F	1.176200	-0.274812	2.306275				
C	1.219464	-1.590253	0.189655				
O	1.610653	-1.449713	-0.999033				
O	0.833321	-2.588828	0.834540				
C	1.733848	1.023386	0.419031				
F	1.476309	2.037413	1.301485				
F	3.081044	1.071880	0.197422				
F	1.144275	1.368640	-0.744289				
**8:2 FTUCA and related structures**							
$CF_3C_6F_{12}CFCHCOO^-$				$CF_3C_6F_{12}CFCH\cdot$			
C	-3.433768	1.177022	-0.405338	C	-3.438529	1.213156	-0.389485
C	-2.786702	0.832351	0.955632	C	-2.786959	0.855952	0.966564
C	-1.231842	0.794910	1.019352	C	-1.232690	0.791603	1.006520
C	-0.535374	-0.142233	-0.002932	C	-0.561665	-0.162174	-0.017354
C	0.945252	-0.466156	0.361201	C	0.915237	-0.503362	0.336934
C	1.763858	-0.990676	-0.847311	C	1.723781	-1.048413	-0.869441
C	3.100975	-1.715462	-0.522767	C	3.062430	-1.759998	-0.519000

C	4.091285	-0.940290	0.273897	C	4.038930	-0.928104	0.285605
F	3.609113	-2.042958	-1.749448	F	3.627654	-2.074924	-1.715061
F	2.750652	-2.886764	0.104925	F	2.774626	-2.908423	0.140956
F	0.958723	-1.855826	-1.535142	F	0.952463	-1.936753	-1.541574
F	2.031971	0.066450	-1.649947	F	2.019112	-0.009277	-1.685340
F	1.515489	0.669206	0.822102	F	1.532473	0.621560	0.775688
F	0.910607	-1.390836	1.345701	F	0.905859	-1.421929	1.325893
F	-1.237261	-1.299550	-0.064923	F	-1.260190	-1.317363	-0.071880
F	-0.596596	0.474736	-1.210101	F	-0.599741	0.442095	-1.230234
F	-0.926452	0.392302	2.275949	F	-0.904538	0.382328	2.256049
F	-0.789625	2.057311	0.835197	F	-0.756720	2.039578	0.808890
F	-3.180601	1.790263	1.832054	F	-3.146276	1.814986	1.851647
F	-3.283182	-0.356986	1.362667	F	-3.285939	-0.330227	1.375296
F	-4.744627	1.399972	-0.215923	F	-4.740875	1.450121	-0.197054
F	-2.883691	2.278726	-0.929270	F	-2.868565	2.306716	-0.911037
F	-3.299806	0.163192	-1.268328	F	-3.309514	0.196491	-1.252103
C	5.205568	-0.374678	-0.177916	C	5.092889	-0.335004	-0.212598
F	3.654435	-0.827743	1.565329	F	3.686821	-0.861678	1.584590
H	5.417847	-0.510358	-1.234442	H	5.586172	-0.221032	-1.162253
C	6.169938	0.536928	0.622007				
O	6.229466	1.678135	0.110099				
O	6.739796	0.024397	1.602822				
$CF_3C_6F_{12}CF\cdot$				$\cdot CHCOO^-$			
C	-3.451071	1.193041	-0.385596	C	5.528192	-0.545681	-0.245138
C	-2.795427	0.836669	0.968799	H	5.144957	-0.298370	-1.246224
C	-1.240577	0.791654	1.007775	C	6.206476	0.462088	0.626890
C	-0.557893	-0.148007	-0.021227	O	6.249793	1.584385	0.025013

C	0.923608	-0.468745	0.325511	O	6.633197	0.152002	1.762030
C	1.737259	-1.004293	-0.882541				
C	3.061000	-1.742610	-0.507282				
C	4.002800	-0.973534	0.359894				
F	3.691149	-1.998804	-1.679035				
F	2.718052	-2.926620	0.075938				
F	0.966508	-1.869233	-1.584516				
F	2.061919	0.042667	-1.673189				
F	1.529300	0.662655	0.764663				
F	0.939242	-1.386945	1.317592				
F	-1.238769	-1.313163	-0.079787				
F	-0.602764	0.461264	-1.231219				
F	-0.905761	0.378333	2.254581				
F	-0.779576	2.046135	0.817594				
F	-3.166306	1.785816	1.859486				
F	-3.278621	-0.358291	1.370818				
F	-4.755959	1.413400	-0.192408				
F	-2.893594	2.295654	-0.901404				
F	-3.309061	0.181942	-1.252805				
F	3.747376	-0.718027	1.620934				

References

1. Goldstein, S.; Rabani, J., The ferrioxalate and iodide–iodate actinometers in the UV region. *J. Photochem. Photobiol. A.* **2008,** *193*, (1), 50-55.
2. Zhao, J.; Shang, C.; Yin, R., A High-Radical-Yield Advanced Oxidation Process Coupling Far-UVC Radiation with Chlorinated Cyanurates for Micropollutant Degradation in Water. *Environ. Sci. Technol.* **2023**.
3. Neese, F., The ORCA program system. *Wiley Interdiscip. Rev. Comput. Mol. Sci.* **2012,** *2*, (1), 73-78.
4. Grimme, S.; Ehrlich, S.; Goerigk, L., Effect of the damping function in dispersion corrected density functional theory. *J. Comput. Chem.* **2011,** *32*, (7), 1456-1465.
5. Grimme, S.; Antony, J.; Ehrlich, S.; Krieg, H., A consistent and accurate ab initio parametrization of density functional dispersion correction (DFT-D) for the 94 elements H-Pu. *Chem. Phys.* **2010,** *132*, (15), 154104.
6. Marenich, A. V.; Cramer, C. J.; Truhlar, D. G., Universal solvation model based on solute electron density and on a continuum model of the solvent defined by the bulk dielectric constant and atomic surface tensions. *J. Phys. Chem. B.* **2009,** *113*, (18), 6378-6396.
7. Krishnan, R.; Binkley, J. S.; Seeger, R.; Pople, J. A., Self-consistent molecular orbital methods. XX. A basis set for correlated wave functions. *Chem. Phys.* **1980,** *72*, (1), 650-654.
8. Clark, T.; Chandrasekhar, J.; Spitznagel, G. W.; Schleyer, P. V. R., Efficient diffuse function-augmented basis sets for anion calculations. III. The 3-21+ G basis set for first-row elements, Li–F. *J. Comput. Chem.* **1983,** *4*, (3), 294-301.
9. Frisch, M. J.; Pople, J. A.; Binkley, J. S., Self-consistent molecular orbital methods 25. Supplementary functions for Gaussian basis sets. *Chem. Phys.* **1984,** *80*, (7), 3265-3269.
10. Hehre, W. J.; Ditchfield, R.; Pople, J. A., Self—consistent molecular orbital methods. XII. Further extensions of Gaussian—type basis sets for use in molecular orbital studies of organic molecules. *Chem. Phys.* **1972,** *56*, (5), 2257-2261.
11. Francl, M. M.; Pietro, W. J.; Hehre, W. J.; Binkley, J. S.; Gordon, M. S.; DeFrees, D. J.; Pople, J. A., Self-consistent molecular orbital methods. XXIII. A polarization-type basis set for second-row elements. *Chem. Phys.* **1982,** *77*, (7), 3654-3665.
12. Bentel, M. J.; Yu, Y.; Xu, L.; Li, Z.; Wong, B. M.; Men, Y.; Liu, J., Defluorination of per-and polyfluoroalkyl substances (PFASs) with hydrated electrons: structural dependence and implications to PFAS remediation and management. *Environ. Sci. Technol.* **2019,** *53*, (7), 3718-3728.
13. Hori, H.; Hayakawa, E.; Einaga, H.; Kutsuna, S.; Koike, K.; Ibusuki, T.; Kiatagawa, H.; Arakawa, R., Decomposition of environmentally persistent perfluorooctanoic acid in water by photochemical approaches. *Environ. Sci. Technol.* **2004,** *38*, (22), 6118-6124.
14. Jing, C.; Zhang, P.-y.; Jian, L., Photodegradation of perfluorooctanoic acid by 185 nm vacuum ultraviolet light. *J. Environ. Sci.* **2007,** *19*, (4), 387-390.
15. Van Hoomissen, D. J.; Vyas, S., 1, 2-Fluorine radical rearrangements: Isomerization events in perfluorinated radicals. *J. Phys. Chem. A* **2017,** *121*, (45), 8675-8687.
